# The Effectiveness Based on Optimal Dose and Administration Route, and Safety Profiles of Stem Cells and Derived Products in the Treatment of Patients With Amyotrophic Lateral Sclerosis: A Systematic Review and Meta‐Analysis

**DOI:** 10.1155/sci/4122493

**Published:** 2026-08-05

**Authors:** Mahdieh Pourasghari, Soraya Babaie, Raha Markazi-Movaghar, Saba Salehpour, Bina Eftekharsadat, Azizeh Farshbaf-Khalili

**Affiliations:** ^1^ Faculty of Medicine, Tabriz University of Medical Sciences, Tabriz, Iran, tbzmed.ac.ir; ^2^ Physical Medicine and Rehabilitation Research Center, Aging Research Institute, Tabriz University of Medical Sciences, Tabriz, Iran, tbzmed.ac.ir

**Keywords:** amyotrophic lateral sclerosis, exosomes, motor neuron disease, neuromuscular disorders, secretome, stem cells, systematic review

## Abstract

**Background:**

This systematic review and meta‐analysis aimed to evaluate the effectiveness of stem cell therapies for patients with amyotrophic lateral sclerosis (ALS) based on optimal dosing and administration routes, as well as the safety profiles of stem cells and their derived products.

**Methods:**

The review followed PRISMA guidelines and involved a comprehensive literature search up to October 2025, receiving ethical approval from Tabriz University of Medical Sciences and registration in PROSPERO. It utilized international databases, including PubMed/MEDLINE, Embase, Cochrane Library, Scopus, Web of Science, ProQuest, ClinicalTrials.gov, and Science Direct. The included studies comprised randomized controlled trials (RCTs), quasi‐experimental studies, and other interventional designs involving ALS patients treated with stem cell therapies. In total, 31 studies were analyzed, featuring 7 controlled trials with 370 participants and 24 non‐controlled pre–post studies with 460 participants. Heterogeneity was evaluated using *I* ^2^ statistics, and subgroup analyses were conducted based on treatment duration and dosing.

**Results:**

A pooled analysis (treatment group: *n* = 93; control group: *n* = 90) demonstrated a significant attenuation in the progression of disease severity, as measured by the ALS Functional Rating Scale (ALSFRS), in stem cell groups versus controls (weighted mean difference [WMD]: 8.89 95% CI: 4.12–13.67; *p* = 0.0003), which was beneficial for both the ≥10 × 10^6^ and <10 × 10^6^ dose sub‐groups. However, a meta‐analysis of single‐arm studies in two control (pre‐intervention) and intervention phases (*n* = 88) demonstrated no significant difference in progression of ALSFRS between study phases by time: month 3 (WMD: −1.27 (−3.01 to 0.47); *p* = 0.15), month 6 (WMD: −2.69 (−5.62 to 0.25); *p* = 0.07), month 9 (WMD: −1.55 (−3.49 to 0.39); *p* = 0.12), and month 12 (WMD: −7.59 (−13.95 to −1.26); *p* = 0.02). An accelerated decline in forced vital capacity (FVC) was observed during the intervention phase, with statistically significant reductions at month 3 (WMD: −10.91; 95% CI: −16.39 to −5.43; *p* < 0.0001) and month 6 (WMD: −15.97; 95% CI: −28.60 to −3.33; *p* = 0.01) compared with the pre‐intervention control phase. Nevertheless, sensitivity analyses excluding studies involving high‐dose mesenchymal stem cell (MSC) therapies demonstrated that these differences were no longer statistically significant. Moreover, no significant change in progression rate was observed at month 9 (WMD: −8.10 (−18.25 to 2.06); *p* = 0.12). The route of MSCs administration (intrathecal [IT], intramuscular [IM], and intravenous [IV]) had no effect on the results of ALSFRS and FVC, reinforced by sensitivity analyses. Adverse events were mostly mild, with headaches most frequent in high‐dose groups.

**Conclusion:**

Stem cell therapy for ALS appears to be safe, with preliminary evidence suggesting potential therapeutic benefit in slowing disease progression in selected patients. Nevertheless, the existing evidence base remains exploratory, and definitive conclusions regarding clinical effectiveness cannot yet be drawn. Future research should prioritize large‐scale and multicenter RCTs with standardized cell manufacturing protocols and longer follow‐up periods.

## 1. Introduction

Motor neuron diseases (MNDs) are a group of rare neurological disorders that selectively affect motor neurons responsible for controlling voluntary muscles [1]. These conditions impact individuals across all age groups, including children and adults, and although clinical presentations may vary between patients, all MNDs are characterized by movement‐related symptoms, particularly muscle weakness [1, 2]. The most common MNDs include amyotrophic lateral sclerosis (ALS) and spinal muscular atrophy (SMA). ALS typically manifests after the age of 40–50 years and may arise from genetic or idiopathic causes. It is defined by the degeneration and death of upper motor neurons (connecting the brain to the spinal cord) and lower motor neurons (linking the spinal cord to skeletal muscles). As a progressive disorder, ALS leads to muscle atrophy and weakness in various regions of the body. The disease is ultimately fatal due to respiratory failure caused by the weakness of the diaphragm and chest wall muscles. No cure currently exists for ALS, and available therapies focus on symptom management [3, 4]. Hereditary ALS accounts for ~10% of cases and is frequently associated with mutations in genes encoding SOD1, C9orf72, or TDP‐43 [3, 5].

The heterogeneity of neuromuscular diseases (NMDs), coupled with the low prevalence of most conditions, has complicated the identification of underlying etiologies and the development of targeted therapies. Furthermore, treatment options for the majority of these disorders remain limited, often restricted to supportive care that fails to halt disease progression [6]. Despite significant advancements in recent decades across fields such as genetics, pathophysiology, and cellular‐molecular mechanisms, a critical need persists for effective therapies to address neuromuscular pathologies and motor impairments [6, 7]. As noted, existing treatments have thus far only delayed the disease progression. Among potential therapeutic strategies explored over the past decade, the concept of tissue regeneration or repair using stem cell‐derived progenitors has gained prominence.

Multiple cell types with diverse tissue origins and characteristics, including myogenic stem/progenitor cells, stromal cells, and pluripotent stem cells, have been investigated over the years, with recent clinical trials yielding variable outcomes. Stem cells are indispensable to living organisms, exhibiting two defining properties: self‐renewal and differentiation potential. Based on potency, they are classified into totipotent, pluripotent, multipotent, oligopotent, and unipotent subtypes. Among these, pluripotent stem cells have been extensively utilized for diverse applications [8]. To date, these stem cells have been employed in clinical trials to assess their safety, efficacy, and therapeutic potential for treating numerous disorders, including Duchenne muscular dystrophy (DMD), SMA, and various neuropathies [9–11].

A 2016 systematic review and meta‐analysis evaluated the impact of cell therapy on survival in rat models and patients with ALS, incorporating preclinical and retrospective clinical studies. Preclinical studies demonstrated a mean survival difference of 79.9 days in favor of the cell‐treated rats. However, the review concluded that the number of clinical trials assessing efficacy was insufficient, with existing studies primarily indicating an absence of serious adverse events. Due to the retrospective design, heterogeneity, and suboptimal quality of clinical data, results were deemed to warrant cautious interpretation [12].

Another systematic review in 2021 assessed multiple stem cell transplantation approaches for ALS across phase I/II clinical trials, yielding inconclusive outcomes. This study conducted a meta‐analysis of therapeutic trials reporting patient outcomes before and after cell administration. Eleven studies involving 220 patients treated with mesenchymal stem cells (MSC, *n* = 152), neural cells (*n* = 57), or mononuclear cells (MNCs: CD34+, CD117+, and CD133+; *n* = 11) were included. Analysis revealed that intraspinal administration of mesenchymal stromal cells (MSCs) led to transient improvements in clinical progression. However, respiratory decline, particularly in forced vital capacity (FVC), was observed across all interventions. Notably, the authors emphasized the need for rigorous preclinical evaluation of stem cell delivery routes prior to advancing to clinical trials [13]. This meta‐analysis combined single‐arm and controlled studies, though the majority of clinical trials were single‐arm.

Given the limited therapeutic options for patients with motor impairments and the profound impact of these conditions on the quality of life for both patients and caregivers, research into novel treatment modalities remains critical. Recent studies on regenerative medicine approaches have shown promise, yet systematic reviews to date have not comprehensively evaluated optimal dosing, delivery methods, or safety profiles of stem cells and their derivatives (e.g., exosomes and secretome) for ALS. Furthermore, conclusive meta‐analyses focusing exclusively on controlled clinical trials are lacking. To address these gaps, this systematic review was designed to determine therapeutic effects with emphasis on appropriate dosing and safety of various stem cell types in ALS by synthesizing data from controlled clinical trials and conducting meta‐analyses of such trials.

## 2. Methods

This systematic review followed methodological standards outlined in the Cochrane Handbook for Systematic Reviews of Interventions [14] and adhered to PRISMA reporting guidelines [15, 16]. The protocol received ethical approval from Tabriz University of Medical Sciences (IR.TBZMED.REC.1403.933) and was prospectively registered in PROSPERO (CRD420251138395). A comprehensive, time‐unrestricted literature search spanning PubMed, Scopus, and Web of Science was conducted through October 2025. Study selection and analysis were structured using the Population, Intervention, Comparison, Outcome, and Study Design (PICOS) framework to define inclusion/exclusion criteria and research questions. Methodological rigor emphasized protocol transparency, ethical compliance, and systematic evidence synthesis aligned with best practices in interventional research reviews.

### 2.1. PICOS

The PICOS framework guided the study question and inclusion and exclusion criteria.

#### 2.1.1. Population

Included: Human participants diagnosed with ALS of any phenotype or disease stage; diagnosis based on El Escorial/Awaji criteria or investigator‐defined clinical criteria; no restrictions on age, sex, geography, or care setting; studies must enrolled ALS patients receiving stem cell–based therapies or stem‐cell–derived products (e.g., exosomes/secretome); Trials must report at least one ALS‐relevant clinical outcome (e.g., ALSFRS, muscle strength, quality of life) and/or safety data.

Excluded: Studies involving animal models or in vitro experiments; patients with neuromuscular disorders other than ALS (e.g., SMA, muscular dystrophies, peripheral neuropathies) unless ALS‐specific results are reported separately; studies without extractable data on dose, safety, or clinical outcomes in ALS patients; case reports, case series, narrative reviews, editorials, or conference abstracts without full data.

#### 2.1.2. Interventions

Included: Interventions including any type of stem cell therapy (e.g., MSCs, neural stem cells, and pluripotent stem cells) or stem‐cell–derived products (such as exosomes or secretome) administered by any route (intravenous [IV], intrathecal [IT], intraspinal, or other); both autologous and allogeneic cell sources were eligible.

Excluded: Preclinical studies involving animal models or in vitro experiments; Interventions using non‐stem‐cell–based therapies (e.g., small molecules, conventional drugs, and physical therapy) unless combined with stem cells and data cannot be separated; Studies without specific information on stem cell dose, safety, or clinical outcomes in ALS patients; Narrative reviews, editorials, commentaries, or conference abstracts lacking primary data.

#### 2.1.3. Comparators or Controls

Included: Eligible comparators included placebo or sham procedures, standard treatments for ALS (e.g., Riluzole, Edaravone, or supportive care), or no intervention. Studies that compare different types, routes, or doses of stem cell therapies against each other could also be included.

Excluded: Studies lacking a comparator or control group unless pre–post data in ALS patients were reported; Comparisons with non‐ALS populations or with interventions unrelated to ALS management (e.g., rehabilitation only, non‐stem‐cell pharmacological agents without standard care); Case reports, case series, or narrative reviews without defined comparisons.

#### 2.1.4. Outcomes

Primary outcomes: To determine the optimal dose and safety of stem cells and stem‐cell–derived products in the treatment of ALS.

Secondary outcomes: To evaluate the effectiveness of stem cell therapies on muscle strength, functional outcomes (e.g., ALSFRS), or disability, and quality of life in patients with ALS.

#### 2.1.5. Study Design

Included: Randomized controlled trials (RCTs), quasi‐experimental studies, semi‐experimental studies, and before–after intervention studies that evaluated stem cell therapies or stem‐cell–derived products in patients with ALS. Both parallel group and crossover designs were eligible.

Excluded: Animal studies, in vitro experiments, narrative reviews, systematic reviews, meta‐analyses, case reports, case series with fewer than five patients, editorials, commentaries, conference abstracts without full data, and study protocols.

### 2.2. Search Strategy

Two independent researchers (Soraya Babaie and Azizeh Farshbaf‐Khalili) systematically searched the following databases up to October 2025: PubMed/MEDLINE, Embase, Scopus, Web of Science, and ClinicalTrials.gov. Persian databases, including Irandoc, SID, and Magiran, were also searched.

Search terms: MeSH keywords and Boolean operators (AND/OR) combining: Lower motor neuron weakness OR Weakness OR Neuromuscular weakness OR Congenital structural myopathies OR Myopathy OR Hereditary myopathy OR Myopathies OR Spinal muscular atrophy OR Amyotrophic lateral sclerosis OR Progressive muscular atrophy OR Motor neuron disease AND Stem cells OR Exosomes OR Secretome AND Clinical trial (see Supporting Information for full strategy).

There were no language and search date restrictions. Other methods of identifying studies comprised reference list checking, Gray literature sources (unpublished studies, reports), and Persian‐language databases: Magiran, SID, IranDoc, IranMedex, Google Scholar, ScienceDirect, and ProQuest.

### 2.3. Study Selection

Search results were imported into EndNote X8 for duplicate removal. Two reviewers (Azizeh Farshbaf‐Khalili and Soraya Babaie) independently screened titles/abstracts, followed by a full‐text assessment for eligibility. Discrepancies were resolved through discussion or consultation with a third reviewer (Bina Eftekharsadat).

### 2.4. Data Extraction

Three independent reviewers (Mahdieh Pourasghari, Saba Salehpour, and Raha Markazi‐Movaghar) systematically extracted data from eligible studies. Relevant information was recorded in a standardized electronic form encompassing authors, publication year, country of origin, study design, objectives, outcome measures, sample size, intervention/control group specifications, follow‐up duration, key findings, and reported adverse events (summarized in Table 1).

**Table 1:** Characteristics of the included studies.

Study ID	Authors	Country	Type of disease	Study design	Participants (e.g., number of participants, age, gender)	Intervention details (e.g., SC dosage, duration of supplementation)	Comparator	Dose of SCs	Source of SCs	Route of administration	Outcomes assessed (both primary and secondary outcomes)	Results data (e.g.,*p* value, confidence interval)	Adverse events and safety
1	Oh et al. [17]	Korea	ALS	Phase I Open‐Label Clinical Trial (non‐randomized, single‐arm).	8 patients (1 died), 3 male and 4 female 45.7 6 10.4 years was the mean age of 7 patients	Bone Marrow Harvest: Under local anesthetic, two extractions (about 50 mL each) are made from the posterior iliac crest. MSC Setup: After being separated by Ficoll density centrifugation, the cells were grown in vitro for 28 days. tested for sterility and surface markers (CD29, CD44, CD73, and CD105). Management: Intrathecal (spinal canal injection at L2−4) is the route. Dosage: 2 injections spaced 26 days apart, each containing 1 × 10^6^ cells/kg.	No control group	1 × 10^6^ cells/kg per dose × 2 injections	BM aspirates from the posterior superior iliac crest under local anesthesia.	Intrathecal	Primary: Safety (adverse events, SAEs). Secondary: Disease progression (ALSFRS‐R, AALS scores, FVC) and CSF cytokine changes (IL‐10, TGF‐β, IL‐6, MCP‐1).	Safety: No severe MSC‐related AEs; 36 mild AEs (e.g., back pain, fever) resolved quickly. 1 SAE (death pre‐treatment due to ALS progression, unrelated to therapy). Disease Progression: ALSFRS‐R decline slowed: Pre‐treatment: −1.38 points/month → Post‐treatment: −0.04 points/month (*p* < 0.001). AALS score decline slowed: Pre‐treatment: 3.10 points/month → Post‐treatment: 0.96 points/month (*p* = 0.002). FVC decline: No significant change (*p* = 0.64). Biomarkers: Anti‐inflammatory cytokines increased (IL‐10, TGF‐β1–3, IL‐6). Pro‐inflammatory MCP‐1 decreased.	Most common: Transient back pain (4 patients), fever (3 patients), headache. All adverse effects were mild (grade I/II) and resolved without intervention.
2	Nabavi et al. [18]	Iran	ALS	Interventional experimental study, phase 1	14 patients: 6 in IV group, 8 in IT group aged 24–60 years 5 female 9 male	Bone Marrow Harvest: Source: 100 mL of BM per patient from the posterior superior iliac crest. MSC Manufacturing: expanded in culture (1–2 passages) after being isolated using a Ficoll gradient. examined for viability and CD44, CD73, CD90, and CD105 markers. Management: Intravenous or intrathecal (IT) routes are available. In 5 milliliters of saline and 2% human serum albumin, the dosage is 2 × 10^6^ cells/kg.	No control group	2 × 10^6^ cells/kg (in 5 mL saline + 2% human serum albumin).	Posterior superior iliac crest (100 mL BM per patient)	Intravenous and intrathecal	Primary: Safety (adverse events, serious complications). Secondary: Disease progression (ALS‐FRS, FVC) and MRI findings.	Safety: Minor AEs: One instance of temporary hypotension (treated with saline) occurred in the IV group. IT group: two incidences of nausea and headache that were treated with NSAIDs. No MRI abnormalities or SAEs due to therapy. One death (respiratory infection, unrelated to therapy) occurred in the IV group. Progress of the Disease: ALS‐FRS: No difference between IV and IT groups (*p* = 0.269), significant decrease over time (*p* < 0.001). FVC: No treatment impact (*p* = 0.731), worsened over time (*p* < 0.001). Functional deterioration was progressive in both groups (ALS‐FRS ↓, FVC ↓). Characterization of Cells: MSCs lacked the hematopoietic markers CD34 and CD45 and expressed the mesenchymal markers CD44, CD73, CD90, and CD105.	Common: headache/nausea (IT), mild hypotension (IV). Severe: two patients needed tracheostomies or PEGs because their ALS was getting worse (unrelated to MSC therapy).
3	Gotkine et al. [19]	Israel	ALS	Phase I/IIa, open‐label, dose‐escalating clinical trial	10 patients (5 in each group) 9 male and 1 female age in group low dose (group A): 63 (4.9) age in high dose (group B): 61 (6.2)	AstroRx Cell Administration: Single intrathecal injection via lumbar puncture (LP). Dosage: Group A: 100 × 10^6^ cells (low dose). Group B: 250 × 10^6^ cells (high dose). Formulation: Cells suspended in 5 mL PlasmaLyte. Immunosuppression: Mycophenolate Mofetil (MMF): 1 g twice daily, starting 2 days pre‐injection and continuing for 1 month post‐injection.	No control group	Group A: 100 × 10^6^ cells (low dose). Group B: 250 × 10^6^ cells (high dose).	AstroRx derived from human embryonic stem cells	Intrathecal (spinal canal injection via lumbar puncture)	Primary: Safety (adverse events, SAEs, lab/imaging abnormalities). Secondary: Efficacy via ALSFRS‐R, SVC, HHD, and serum NfL biomarker.	86 TEAEs [treatment‐emergent adverse events] (63 mild, 19 moderate, 4 severe); 9 SAEs [serious adverse events] (e.g., post‐LP headache requiring blood patch). No AstroRx‐related SAEs or CNS abnormalities on MRI/CT. 3 deaths (respiratory failure from ALS progression; unrelated to treatment). Efficacy: ALSFRS‐R: Group A (100M cells): 66% slower decline at 3 months (*p* = 0.039); no sustained benefit at 6/12 months. Group B (250M cells): 45% slower decline at 3 months (*p* = 0.002); effect faded by 6 months. Combined Groups: 53% attenuation in decline at 3 months (*p* < 0.001). SVC: Accelerated decline post‐treatment (*p* = 0.01). HHD/Grip Strength: No significant improvement (*p* = 0.24). Biomarkers (NfL): No meaningful changes.	Procedure‐Related: Post‐LP headache (50%), injection site pain (30%), back pain (10%). Immunosuppression (MMF): Mild nausea, anemia (resolved). No treatment‐specific toxicity.
4	Ruiz‐Navarro and Kobinia [20]	Austria	ALS	Open label pilot study	Number: 85 ALS patients (57 men and 28 women). Age: 20–80 years	Intrathecal (IT): 5–10 mL (mean 7.9 ± 2.9 mL), containing 5.7 ± 5.6 million CD34+ cells. Intramuscular (IM): 2–8 mL (mean 4.8 ± 1.2 mL), containing 3.6 ± 3.6 million CD34+ cells. Intravenous (IV): 1–8 mL (mean 2.3 ± 1.3 mL), containing 1.5 ± 1.6 million CD34+ cells	No control group	Routes: Intrathecal (IT): 5–10 mL BM‐MNCs. Intramuscular (IM): 2–8 mL BM‐MNCs. Intravenous (IV): 1–8 mL BM‐MNCs + 20–40 mL BM plasma (BMP) BM‐MNCs: Adapted to patient weight and extracted BM volume (no set cell count). CD34+ Cells: Measured via laboratory analysis in accordance with ISHAGE recommendations.	Autologous bone marrow: patient‐derived MNCs and plasma	Intrathecal Intramuscular Intravenous	Primary Outcome: Safety: Point‐of‐Care Stem Cell Therapy (POCST)‐related incidence of adverse events (AEs) and serious adverse events (SAEs). Secondary Outcomes: Impairment of Function: measured using the ALS‐FRS‐R, or ALS Functional Rating Scale‐Revised.	Functional Decline (ALSFRS‐R): Mean Decline: 3.8 points post‐treatment (95% CI: 1.1–6.2); no improvement observed. 52.6% stabilized temporarily; 47.3% worsened. Key Correlations: Higher pre treatment ALSFRS‐R linked to shorter diagnosis to treatment time (*p* = 0.002). Lower post treatment scores associated with elevated BM CD34+ cells (*p* = 0.038) and leukocytes (*p* = 0.010). Survival Outcomes: Post Diagnosis: Mean survival: 99.3 months (95% CI: 78.6–119.9). Survival rates: 96% at 6 months, 57% at 10 years. Post Treatment: Median survival: 17 months (95% CI: 11.4–22.5). Survival rates dropped to 37.2% by 24–45 months. Risk Factors: Male sex (*p* = 0.047), high BM CD34+ cells (*p* = 0.021), lack of suboccipital/IM infusions (*p* < 0.001). Bone Marrow (BM) Analysis: BM Extraction: Higher BM volume (>130 mL) linked to lower pre separation CD34+ cells (*p* = 0.025) but higher post separation CD34+ (*p* = 0.025). Younger age correlated with more BM harvested (*p* = 0.025). CD34+ Cells: Higher IM CD34+ doses (>4 million) associated with better ALS‐FRS‐R (*p* = 0.002). Vitality and Leukocytes: BM Vitality: Higher pre‐separation vitality (>80%) reduced vitality loss (*p* = 0.001) and improved post‐treatment ALS‐FRS‐R (*p* = 0.009). Leukocytes: Elevated preseparation leukocytes (>12/nL) linked to poorer outcomes (*p* = 0.004).	Most Common: Headache (40% of patients) post lumbar puncture, resolved with standard care. No Major Side Effects: No severe or long term complications reported.
5	Petrou et al. [21]	Israel	ALS	Phase 1/2 and 2a Clinical Trials	Number: 26 ALS patients (12 in Phase 1/2; 14 in Phase 2a). Age: 20–75 years with definite/probable ALS (El Escorial criteria), disease duration <2 years. Groups: Phase 1/2: IM group: 6 patients (ALS‐FRS‐R >30). IT group: 6 patients (ALS‐FRS‐R <30, FVC >50%). Phase 2a: 14 patients (ALS‐FRS‐R >30, FVC ≥50%) received combined IT + IM therapy.	Cell Therapy: MSC‐NTF Cells: Autologous bone marrow‐derived mesenchymal stem cells (MSCs) differentiated into neurotrophic factor‐secreting cells using growth factors (e.g., fibroblast growth factor, platelet‐derived growth factor). Dosage: Phase 1/2: IM: 24 × 10^6^ cells (1 × 10^6^/site at 24 arm muscle sites). IT: 1 × 10^6^ cells/kg. Phase 2a: Low: 1 × 10^6^ cells/kg IT + 24 × 10^6^ cells IM. Mid: 1.5 × 10^6^ cells/kg IT + 36 × 10^6^ cells IM. High: 2 × 10^6^ cells/kg IT + 48 × 10^6^ cells IM.	No control group	Phase 1/2: IM: 24 × 10^6^ cells total (1 × 10^6^ cells/site at 24 sites in arm muscles). IT: 1 × 10^6^ cells/kg. Phase 2a (Combined IT + IM): Low Dose: 1 × 10^6^ cells/kg IT + 24 × 10^6^ cells IM. Mid Dose: 1.5 × 10^6^ cells/kg IT + 36 × 10^6^ cells IM. High Dose: 2 × 10^6^ cells/kg IT + 48 × 10^6^ cells IM.	Autologous bone marrow: patient‐derived MSCs	Intramuscular (IM): Biceps and triceps muscles of the arms. Intrathecal (IT): Injection into the spinal canal.	Primary Outcomes: Safety and Tolerability: The frequency of adverse events (AEs) following administration, such as pain, infection, or neurological problems, are the main outcomes. Secondary Outcomes: 1. Decline in Function: 2. ALS‐FRS‐R: Functional score change rate. 3. Forced Vital Capacity (FVC): The ability to breathe. 4. Preservation of Muscles: 3D MRI is used to measure muscle mass. 5. Changes in Electrophysiology: 6. Compound Muscle Action Potential (CMAP): Bicep muscle amplitude recorded.	ALS‐FRS‐R and FVC Progression: Phase 1/2 (IT Cohort): ALS‐FRS‐R decline improved from −1.56/month to +0.28/month; FVC decline slowed from −3.5%/month to −2.3%/month. Phase 2a (Combined IT + IM): ALS‐FRS‐R decline improved from −1.4/month to −0.6/month (*p* = 0.052); FVC improved from −2.6%/month to + 0.86%/month (*p* = 0.036). Responder Analysis (IT/IT + IM): 87% (13/15 patients) showed ≥25% improvement in ALS‐FRS‐R/FVC at 6 months; 67% (10/15) showed ≥50% improvement. Electrophysiology (CMAP): Non‐significant trends toward improved CMAP amplitudes in injected arms (no *p*‐values reported). Muscle Volume (MRI): Slower atrophy in injected vs. non‐injected arms (Phase 2a), but not statistically significant. Immunologic Response: Upregulation of regulatory T‐cells (CD4+CD25+) post‐treatment (trend only; no *p*‐values). FVC improvement: *p* = 0.036 (significant). ALS‐FRS‐R trend: *p* = 0.052 (near‐significant).	Headache (13), fever (11), back/leg pain (8), vomiting (3), neck stiffness (2), general weakness (1), bruising (1), spasticity (1)
6	Syková et al. [22]	Czeck republic	ALS	A single‐center, prospective, open‐label study, without a placebo control group	23 patients mean age: 51.2 ± 1.7 12 female and 11 male	Cell Therapy: Autologous bone marrow‐derived mesenchymal stem cells (BM‐MSCs). To assess the rate of pretreatment illness progression, the patients underwent three neurological examinations at 6, 3, and 1 months (±1 week) before to BM‐MSC application (prescreening period). In order to evaluate the safety and effectiveness of the treatment, there were regular intervals between examinations at 3, 6, 9, 12, and 18 months over the follow‐up period.	No control group	15 ± 4.5 × 10^6^ BM‐MSCs per dose.	Autologous bone marrow harvested from the posterior iliac crest under local anesthesia.	Intrathecal	Primary Outcome: Safety: Adverse Events (AEs): Long‐term hazards (tumor growth, abnormal MRI) and immediate/local reactions (pain, infection, paralysis). Monitoring: Brain/spinal MRI at 12 months after therapy, lab tests (blood count, renal/hepatic function), and vital signs. Secondary Outcomes: Progress of the Disease: Rate of decrease before and after treatment, as measured by the ALS Functional Rating Scale (ALSFRS). FVC: The capacity of the respiratory system. Muscle strength (0–5) is measured by the Muscle Weakness Scale (WS). Analysis of Statistics: ALSFRS decrease slopes before therapy (3–6 months baseline) and after treatment (3, 6, 9, 12, and 18 months) were compared using a paired *t*‐test.	ALSFRS Decline: Overall (*n* = 23): Significant slowdown at 3 months (*p* < 0.02) and 6 months (*p* < 0.05). Fast‐Progressing Subgroup (*n* = 12): Marked slowdown at 3 months (*p* < 0.001) and 6–12 months (*p* < 0.01). Stable Pre‐Treatment Group: No significant effect. FVC: 80% maintained ≥70% FVC at 9 months; 60% at 12 months. Muscle Weakness (WS): 75% stable at 3 months; gradual decline by 12 months.*p*‐values: ALSFRS: *p* < 0.02 (3 months), *p* < 0.05 (6 months), *p* < 0.001 (subgroup analysis). No *p*‐values reported for FVC/WS stability (descriptive data only). Confidence Intervals: Not explicitly provided in the text.	Most Common: Headache (30% of patients) post treatment, resembling post lumbar puncture pain (resolved without intervention). Serious Events: 1 death due to respiratory failure (unrelated to treatment). No treatment‐related severe complications (e.g., tumors, spinal pathology on MRI)
7	Petrou et al. [23]	Israel	ALS	A single center open, phase II clinical trial	20 patients, 16 male, 4 female, mean age: 49.97	Treatment: Repeated intrathecal (IT) injections of autologous mesenchymal stem cells (MSCs). Frequency: 1–4 injections per patient, administered every 3–6 months over up to 2 years.	No control group	Per Injection: 1 × 10^6^ cells/kg body weight	150 mL of autologous bone marrow was extracted from patients while they were under mild general anesthesia.	Intrathecal	Primary: Safety of repeated intrathecal MSC injections (adverse events). Secondary: ALSFRS‐R decline (disease progression). FVC changes (respiratory function). Kaplan–Meier survival analysis (time to ALSFRS‐R progression >2 points).	ALSFRS‐R Progression: Overall: Disease progression slowed significantly (*p* = 0.0038): Pre‐treatment decline: −1.054 ± 0.86/month → Post‐treatment: −0.445 ± 0.81/month. Response Rates: 79% (15/19 patients) showed >25% improvement after 1st–2nd injection; 80% (8/10) after 3rd–4th. 7/19 patients had ALSFRS‐R score increases (mean + 2.86 points) post‐treatment. Survival Benefit: Delayed progression (>2‐point ALSFRS‐R decline; *p* = 0.049). FVC Trends: Respiratory Decline Slowed: Median monthly FVC decline reduced from −1.32% (pre treatment) to −0.22% post treatment (non‐significant).	Common: Mild headache/back pain (3 patients) post‐lumbar puncture; transient fatigue (1 patient). Serious (Unrelated to Treatment): Aspiration pneumonia (1), ankle fracture (1), renal colic (1). 1 Death: Due to rapid ALS progression (respiratory failure, unrelated to therapy). No treatment‐related severe AEs or long‐term complications.
8	Barrientos. et al. [24]	Spain	ALS	A Phase I/II clinical trial, randomized, double‐blinded, placebo‐controlled trial	22 patients age: 55.6 ± 2.1, range (33–70)	Treatment: Intramuscular (IM) injection of autologous bone marrow mononuclear cells (BMMCs) into the tibialis anterior (TA) muscle. Control: Contralateral TA muscle injected with normal saline (placebo). Randomization: 1:1 ratio (BMMCs vs. placebo), double‐blinded (patients and assessors).	No control group	Bone marrow mononuclear cells (BMMCs): Median 499 × 10^6^ cells (range: 206–1086 × 10^6^ cells) per patient. Volume: 2 mL (split into four 0.5 mL injections per tibialis anterior (TA) muscle).	Autologous Bone Marrow: Harvested from the posterior iliac crest under sedation.	Intramuscular (IM): Injected into the tibialis anterior (TA) muscle of both legs (experimental side: BMMCs; control side: saline).	Safety: Incidence of adverse events (AEs) related to intramuscular BMMC injections. Secondary Outcomes: Functional/Electrophysiological Parameters: Muscle Strength (MRC scale). CMAP peak amplitude/area. Fiber Density (FD). D50 Index (quantifying motor unit recruitment). MUNIX (motor unit number index). MUSIX (motor unit size index).	D50 Index: Improved significantly in BMMC‐treated muscles vs. placebo at 90 days (*p* < 0.01) and 180 days (*p* < 0.01), indicating better motor unit recruitment. Fiber Density (FD): Increased over time in both treated and placebo muscles (*p* < 0.05 at 180 and 360 days), suggesting motor unit remodeling. Muscle Strength (MRC): Declined similarly in both groups by 180 days (*p* < 0.05), showing no treatment benefit. Patient Heterogeneity: Two subgroups identified: Stable CMAP/MUNIX (4 patients, no progression). Progressive decline (5 patients, worsening CMAP/MUNIX).	Number of non‐serious adverse events: 29 Non‐serious adverse events per patient (mean/median/range): 1.8/1/1–5 Number of non‐serious, not‐procedure related events 22 Non‐serious, non‐procedure related events per patient (mean/median/range): 1.4/1/0–4 Number of non‐serious, procedure related events: 7 Non‐serious, procedure related events per patient (mean/median/range): 0.4/0/0–2 Number of serious adverse events: 15 Serious adverse events per patient (mean/median/range): 1.5/1/1–3 Number of non‐serious, non‐procedure related events: 15 Serious, non‐procedure related events per patient (mean/median/range): 1.5/1/1–3 Number of serious, procedure related events: 0 Serious, procedure related events per patient (mean/median/range):ND Patients with >1 non‐serious adverse event: 16 Patients with >1 non‐serious procedure‐related adverse event: 6 Patients with >1 serious adverse event: 10 Patients with >1 serious procedure‐related adverse Event: 0
9	Mazzini et al. [25]	Italy	ALS	Phase 1 clinical trial prospective, open pilot study	6 patients, ages: 30, 57, 54, 35, 67, 38	Transplantation: GMP‐grade fetal human neural stem cells (h NSCs) derived from natural in utero death (miscarriages). Procedure: Microinjections into the anterior horns of the lumbar spinal cord. Unilateral group: 3 patients received 3 unilateral injections. Bilateral group: 3 patients received bilateral injections (3 + 3 microinjections).	No control group	Exact cell numbers per injection not specified, but bilateral patients received ~5× more cells than unilateral.	Fetal human neural stem cells (h NSCs): Sourced from natural miscarriages	Intraspinal microinjections: Delivered directly into the lumbar spinal cord (anterior horns).	Primary outcome: Safety: Adverse events (AEs) related to cells or neurosurgery. Disease progression (ALS‐FRS‐R score). Secondary Outcomes: Functional improvement: ALS‐FRS‐R sub score ambulation.MRC score (e.g., tibialis anterior muscle strength).	Safety: No severe AEs related to treatment. No accelerated disease progression observed over 18 months post‐surgery. Autopsies confirmed deaths were due to disease progression (respiratory failure), not treatment. Functional Outcomes: 2 patients: Transient improvement in ALS‐FRS‐R ambulation sub score (from 1 to 2). 1 patient: Sustained improvement in MRC score for tibialis anterior (7 months). 3 patients: Refused PEG/invasive ventilation; died from respiratory failure at 8 months post‐surgery. Statistical Data: No *p*‐values or confidence intervals reported (phase I trial focused on safety, not efficacy).	No severe treatment‐related AEs. Deaths: 3 patients died due to respiratory failure (disease progression, unrelated to treatment).
10	Shamsaei et al. [26]	Iran	ALS	Single‐center, phase I clinical trial	7 patients: 5 male and 2 female mean age was 44.7 ± 9.01 years.	Treatment: Autologous bone marrow‐derived mesenchymal stem cells (BM‐MSCs) administered intrathecally. Procedure: Single intrathecal injection via lumbar puncture (L2–L3 or L3–L4 levels).	No control group	15 ± 3 × 10^6^ BM‐MSCs per patient.	Autologous Bone Marrow: Harvested from the posterior superior iliac crest under short general anesthesia.	Intrathecal: Delivered into the subarachnoid space via lumbar puncture.	Primary Outcome: Safety: Incidence of adverse events (AEs) related to intrathecal autologous BMMSC (bone marrow derived mesenchymal stem cells) administration (e.g., organ dysfunction, MRI abnormalities). Secondary Outcomes: Efficacy: ALS Functional Rating Scale‐Revised (ALSFRS‐R): Disease progression rate. Forced Vital Capacity (FVC): Respiratory function decline.	Safety: 1 mild headache (resolved with analgesics); no severe AEs or MRI abnormalities. No treatment‐related deaths; 2 excluded patients died from disease progression. Efficacy: ALSFRS‐R decline slowed: Pretreatment: −5.4 ± 2.3 points/10 months → Post treatment: −2.0 ± 3.08 (*p* = 0.014). 65.7% of improvement attributed to treatment. FVC decline slowed: Pretreatment: −12.6 ± 5.22%/10 months → Post treatment: −4.8 ± 14.72% (*p* < 0.001). 43% of improvement linked to therapy. No correlation between ALSFRS‐R and FVC changes (*p* > 0.05).	Patient experienced a mild headache post‐injection (resolved with analgesics).
11	Barczewska et al. [27]	Poland	ALS	Single‐center, open‐label, single‐arm Phase I clinical trial.	43 patients (16 females and 27 males) age: 57.3 ± 6.26 (36–79)	Wharton’s jelly‐derived mesenchymal stem cells (WJ‐MSCs) are injected intrathecally as a treatment. Concurrent Therapy: Riluzole, a common medication for ALS, was administered to all patients at a dose of 100 mg per day. Three shots were given to 14 patients in total. Two doses for 20 individuals. One injection for nine individuals. The time between injections is 2 months.	No control group	Per Injection: 0.42 × 10^6^ cells/kg (average 30 × 10^6^ cells per patient per injection).	Wharton’s Jelly: Derived from human umbilical cords collected after elective C‐sections (gestational age ≥38 weeks).	Intrathecal: Cells delivered into the cervical, thoracic, or lumbar spinal regions (tailored to clinical symptoms).	Primary Outcome: Adverse events (AEs) monitored over 6 months. Secondary Outcomes: Implicitly included tolerability (e.g., headache incidence).	Safety Results: No SAEs (serious adverse events) reported during the 6‐month treatment period. 1 mild AE (adverse events): Headache (2.3% of participants) after the first injection in a 62‐year‐old male (resolved spontaneously). No other AEs (adverse events) (e.g., infection, neurological deficits, or procedural complications) observed. Statistical Data: No *p*‐values or confidence intervals reported (safety‐focused study without efficacy comparisons).	Headache: 1 case (non‐serious, resolved without intervention). Procedural Notes: No post‐puncture syndromes (e.g., cerebrospinal fluid leakage) despite cerebrospinal fluid sampling during injection. Mitigation measures: Patients received intravenous/oral fluids and 3‐h post‐procedure bed rest.
12	Staff et al. [28]	USA	ALS	Single‐center, open‐label, Phase I dose‐escalation safety trial.	27 patients (12 female,15 male) Range of ages (36–70)	Autologous adipose‐derived mesenchymal stromal cells (AD‐MSCs) are infused intrathecally as a treatment. The adipose biopsy procedure. Open fat biopsy of the abdomen ≥8 weeks before to therapy. Cell Processing: In a GMP facility, MSCs are separated, grown, and cryopreserved. Administration: MSCs are administered intrathecally after being suspended in lactated Ringer solution.	No control group	5 Dose‐Escalation Groups: Group 1: 1 × 10^7^ cells (single injection). Group 2: 5 × 10^7^ cells (single injection). Group 3: 5 × 10^7^ cells (2 monthly injections). Group 4: 1 × 10^8^ cells (single injection). Group 5: 1 × 10^8^ cells (2 monthly injections)	Autologous AD‐MSCs (autologous adipose derived mesenchymal stromal cells): Harvested from the patient’s abdominal adipose tissue (via biopsy).	Intrathecal: Delivered via standard lumbar puncture.	Primary: Safety of intrathecal AD‐MSCs (adverse events, MRI abnormalities, tumor formation via autopsy). Secondary: Disease progression (ALSFRS‐R scores) and transient patient‐reported improvements (e.g., limb strength, bulbar function).	Safety: Dose‐dependent adverse events: 80% of patients receiving large doses (1 × 10^8^ cells) have mild back or leg pain that goes away in a few weeks. Changes in CSF: After treatment, there were elevated protein and monocytes, which returned to normal by week four. MRI results: 90% of high‐dose patients have thicker lumbosacral nerve roots. Four patients had autopsies; no arachnoiditis or malignancies were found. Efficacy: ALSFRS‐R: All patients continue to have illness progression. Subjective improvements: 17 out of 29 patients experienced short‐term advantages, such as increased energy and less stiffness.	Most common: Pain (managed with NSAIDs/gabapentin) and transient CSF/MRI changes. No severe or life‐threatening events (e.g., tumors, infections).
13	Glass et al. [29]	Israel	ALS	Multicenter, open‐label, Phase I/II dose‐escalation safety trial	15 patients age: 48.7 (10.0)	Human spinal cord‐derived neural stem cells (HSSCs) are transplanted into both spinal cords as a treatment. For groups A–D, the surgical procedure consists of cervical injections (C3–C5). For group E, lumbar injections (L2–L4) plus cervical injections (two different operations). Immunosuppression: tacrolimus/mycophenolate mofetil (continuous), prednisone (1 month), and basiliximab (perioperative). Cell Preparation: HSSCs separated by 4 mm and suspended in 10 µL per injection.	Control Groups: External Historical Controls (no concurrent control group): Ceftriaxone trial placebo arm (NEALS dataset). Pro ACT dataset (control arms of prior ALS trials). Emory ALS Center database (matched for age, sex, and disease duration).	Dose Escalation: Group A: 10 cervical injections (5 per side). Groups B‐D: 20 cervical injections (10 per side). Group E: 20 lumbar + 20 cervical injections (two surgeries). Total Cells: 2–16 million cells per patient (dose‐dependent). 1/B: 100,000 1/C: 100,000 1/D: 100,000 1/E: 100,000 A: 200,000 B: 400,000 C: 600,000 D: 800,000 E: 800,000	Human Spinal Cord‐Derived Neural Stem Cells (HSSCs): Derived from human spinal cord tissue	Intraspinal: Cervical: Bilateral injections at C3–C5. Lumbar: Bilateral injections at L2–L4 (group E only).	Primary: Safety of intraspinal HSSC transplantation (adverse events, severe complications). Secondary: Disease progression via ALSFRS‐R, FVC, and grip strength.	Safety: 165 adverse events in total (phase 2), primarily related to immunosuppressive side effects (e.g., headache, hyperglycemia) and surgical discomfort. Serious complications: One instance of spinal cord edema necessitated a reoperation; after 5 months, there was a partial recovery. One instance of excruciating, unresponsive neuropathic pain, most likely from the spine. Two pulmonary embolisms (known ALS/surgical hazards); two deaths due to the progression of ALS. 14 AEs are possibly/probably associated to stem cells, but none are conclusively linked. Progress of the Disease: In comparison to historical controls (ceftriaxone, Pro ACT, and Emory datasets), there was no acceleration in ALSFRS‐R, FVC, or grip strength deterioration. Variability: There was no consistent treatment effect, but some subjects advanced more or less quickly.	Blood disorders: 1 GI disorders: 13 infection: 8 injury or procedural complications: 17 Disorders of metabolism and nutrition: 9 Musculoskeletal disorders: 15 Nervous system disorders: 20 Psychiatric disorders: 3 Renal‐urinary disorder: 3 Respiratory disorder: 1 Skin disorder: 3 Vascular disorder: 3 Other: 3
14	Berry et al. [30]	USA	ALS	Randomized, double‐blind, placebo‐controlled, phase 2 trial	48 participants:35 male, 13 female/12 placebo: 10 male, 2 female/36 MSC‐NTF:25 male, 11 female The mean age of the participants: 51.1 /Placebo:53.5/MSC‐NTF:50.3	Main Group: Intervention: Autologous MSC‐NTF cells (NurOwn). Dosage: Intrathecal (IT): 125 × 10^6^ cells (5 mL). Intramuscular (IM): 48 × 10^6^ cells total (24 injections × 2 × 10^6^ cells each). Route: Combined IT (spinal needle) and IM (biceps/triceps). Source: Bone marrow‐derived MSCs (expanded/differentiated to secrete neurotrophic factors).	Placebo (Dulbecco Modified Eagle Medium). Intervention: Placebo (cell culture medium). Route: Identical IT and IM administration (no cells).	Main Group: MSC‐NTF Cells: Dosage: Intrathecal (IT): 125 × 10^6^ cells in 5 mL Intramuscular (IM): 24 injections totaling 48 × 10^6^ cells (likely 2 × 10^6^ cells per injection, given 1 mL syringes).	Bone marrow‐derived MSCs	Main Group: Combined IT (spinal needle) and IM (biceps/triceps) Placebo: Identical IT and IM administration	Primary Outcome: Safety (Frequency of treatment‐related adverse events (TEAEs/SAEs), lab abnormalities, and vital signs.) Secondary Outcomes: 1Efficacy: ALSFRS‐R/SVC Decline Rates Responder Analysis Exploratory Outcomes: 1. Muscle Strength (HHD): Compare injected vs. non‐injected arm. 2. CSF Biomarkers: Changes in neurotrophic factors (e.g., VEGF, GDNF), microRNAs, and CHIT‐1 levels.	ALSFRS‐R Scores: Overall Cohort: Non‐significant trend toward improvement in MSC‐NTF group (e.g., +1.7 vs. −0.4 points/month at week 2; *p* = 0.11). Rapid Progressors Subgroup (fastest disease decline): Significant ALSFRS‐R slope improvement at week 2 (+3.3 vs. −1.3; *p* = 0.021) and week 4 (+2.0 vs. −0.1; *p* = 0.033). Responder Analysis (≥1.5 points/month improvement): Overall: 47% MSC‐NTF vs. 9% placebo responders at week 4 (*p* = 0.033). Rapid Progressors: 80% MSC‐NTF vs. 0% placebo at week 4 (*p* = 0.004; 95% CI: 59.8%–100%). Biomarker Changes in CSF: Neurotrophic Factors (MSC‐NTF group): Increased VEGF (+630 pg/mL; *p* = 0.016), HGF (+107 pg/mL; *p* = 0.004), and LIF (+12 pg/mL; *p* = 0.0008). Inflammatory Markers: Reduced MCP‐1 (−17 pg/mL; *p* < 0.0001), SDF‐1α (−47 pg/mL; *p* = 0.0014), and CHIT‐1 (−1413 pg/mL; *p* = 0.027). Caspase‐3 (apoptosis marker) decreased (−2.1 pg/mL; *p* < 0.0001). Correlations: VEGF inversely correlated with MCP‐1 (*r* = −0.56; *p* = 0.003). Greater MCP‐1 reduction linked to ALSFRS‐R improvement (*p* < 0.05 at weeks 4–24).	Headache and procedural headache Back pain Pyrexia Arthralgia Injection site pain Constipation Pain in extremity Neck pain Myalgia Cough Nausea
15	Oh et al. [31]	Korea	ALS	A parallel‐group, randomized, and con‐ trolled phase 2 trial	59 participants: MSC Group, *n* = 32(18 M /14 F) Control Group, *n* = 27(11 M/16 F)	MSC Group: Intervention: Autologous bone marrow‐derived MSCs (BM‐MSCs). Dosage: 1 × 10^6^ cells/kg × 2 doses (26‐day interval)	Control Group: Intervention: Standard care (Riluzole 100 mg/day + symptomatic treatments). Note: No sham procedures (e.g., bone marrow aspiration) performed for ethical reasons.	1 × 10^6^ cells/kg × 2 doses (26‐day interval)	Patient’s own bone marrow	Intrathecal (via lumbar puncture at L2–L4)	Primary Outcomes: Functional Decline: ALSFRS‐R Score Change: Mean change from baseline (0 months) to 4 months and 6 months post‐treatment. Secondary Outcomes: Disease Progression: ALSFRS‐R Slope: Rate of monthly decline during 6‐month follow‐up vs. 3‐month lead‐in period. Responder Analysis: ≥50% improvement in ALSFRS‐R slope decline at 4/6 months (“good responders”). Other Metrics: Changes in AALS (functional impairment), FVC (respiratory function), and SF‐36 (quality of life) at 4 months. Clinical Interventions: Timing of tracheostomy, non‐invasive ventilation, or feeding tube placement. CSF Biomarkers: Changes in inflammatory cytokines (e.g., IL‐6, MCP‐1, TNF‐α) and correlations with treatment response. Genetic Screening: Exclusion of ALS‐linked mutations (C9orf72, SOD1, FUS, etc.) to control for genetic variability. Long‐Term Survival: Overall survival and tracheostomy‐free survival tracked for up to 75 months post‐trial.	Primary (ALSFRS‐R Score): 4 Months: MSC decline = −1.69 vs. control −4.67 (difference = 2.98; 95% CI: 1.48–4.47; *p* < 0.001). 6 months: MSC vs. control difference = 3.38 (95% CI: 1.23–5.54; *p* = 0.003). Secondary: Slowed Decline: ALSFRS‐R slope decline reduced in MSC group (*p* = 0.003 at 4 months; *p* = 0.018 at 6 months). Responders: ≥50% improvement in ALSFRS‐R decline in 69% (MSC) vs. 19% (control) at 4 months (*p* = 0.002). AALS Scores: Greater improvement in MSC group (−7.18; *p* = 0.009). FVC/SF‐36: No significant differences. Post Hoc Analyses: CSF Biomarkers: Increased: Anti‐inflammatory cytokines (TGF‐β1–3, IL‐6, IL‐10; *p* < 0.05). Decreased: Pro‐inflammatory markers (TNF‐α, MCP‐1; *p* < 0.05). Correlation: TGF‐β1 ↑ linked to MCP‐1 ↓ in responders (*R* ^2^ = 0.259; *p* = 0.011–0.016). Genetics: No mutations in ALS‐linked genes (C9orf72, SOD1, FUS). Survival: No group difference overall (MSC: 55 vs. control: 48 months; *p* = 0.487), but younger MSC patients (<54 years) trended toward longer survival (65 vs. 48 months; *p* = 0.10).	Incidence: No significant difference in overall adverse events (AEs) between MSC (61%–64%) and control groups (71%–74%) at 4/6 months (*p* = 0.36–0.38). Common AEs in MSC Group: Influenza‐like illness (21%), back pain (15%), musculoskeletal pain (15%). Treatment‐Related Reactions: Mild, transient headache, fever, or pain (9% incidence; resolved within 48 h). Serious AEs: MSC group had fewer SAEs (9% vs. 19% in controls); none were treatment‐related. Mortality: 1 death (MSC, respiratory failure) vs. 3 (control); all attributed to disease progression.
16	Feldma et al. [32]	USA	ALS	Phase 1, Open‐Label, First‐in‐Human clinical trial. Nonrandomized	15 subject (13 male/2 female age: 35 to 66 years old) included, 7 subject died	Cervical Intraspinal Injections: Procedure: 5 unilateral injections via a novel spinal‐mounted device. Surgery: C3–C5 laminectomy. Prior Lumbar Injections (Group E): 10 bilateral lumbar injections.	No control group	Per Injection: 100,000 neural stem cells (10,000 cells/μL concentration). Total per Session: Cervical: 500,000 cells (5 injections). Lumbar + Cervical (Group E): Up to 1.5 million cells (10 lumbar + 5 cervical injections).	NSI‐566RSC HSSC (human spinal cord‐derived neural stem cells)	Intraspinal: Cervical: Targeted C3–C5 spinal cord regions. Lumbar: Bilateral injections (prior Group C patients)	Primary Outcomes: Safety: Complications: Minimal perioperative/postoperative issues; no hemorrhage, cysts, or inflammation at injection sites (confirmed by autopsies). Mortality: 7 deaths (6 ALS‐related respiratory failure, 1 cardiac defect); none attributed to treatment. Feasibility: Cervical and dual‐targeted injections were well‐tolerated without accelerating disease progression. Secondary Outcomes: Functional Measures: ALSFRS‐R: Modest improvements or slower decline in some subjects (e.g., Group E: Subjects 11/12 showed improved scores). GST: Strong correlation with ALSFRS‐R, suggesting utility in future trials. FVC/HHD/EIM: Stable or mild declines in most subjects. Disease Progression: Bimodal Benefit: ALSFRS‐R progression rates improved for 6 months post‐surgery and again after second transplants.	Functional Improvements: 50% of subjects had better‐than‐expected outcomes at 6–15 months post‐surgery. Group E: Dual‐targeted transplants linked to delayed progression. Safety: No treatment‐related SAEs; deaths attributed to ALS progression or pre‐existing conditions.	Common: None beyond typical ALS progression. Serious: 7 deaths (6 ALS‐related, 1 unrelated cardiac defect). No evidence of transplant‐related pathology (e.g., inflammation, cysts).
17	Glass et al. [33]	USA	ALS	Phase I, Open‐Label, Non‐Randomized clinical trial. Single‐Arm	12 ALS patients male (range of age: 37−62)	Intraspinal Injections: Target: Lumbar spinal cord (vertebral levels T11–T12). Dosage: Per Injection: 100,000 neural stem cells (10,000 cells/μl concentration). Total per Patient: Unilateral: 5 injections (500,000 cells total). Bilateral: 10 injections (1,000,000 cells total). Procedure: Cells administered via a spinal‐mounted stabilization device during a laminectomy.	No control group	Dosage: Per Injection: 100,000 neural stem cells (10,000 cells/μL concentration). Total per Patient: Unilateral: 5 injections (500,000 cells total). Bilateral: 10 injections (1,000,000 cells total).	Cell Line: NSI‐566RSC (human fetal‐derived neural stem cells). Origin: Isolated from the spinal cord of a single 8‐week‐old fetus.	Cells injected into the spinal cord parenchyma	Primary Outcomes: Safety: Surgical Complications: 1 dural fistula (repaired), 1 wound dehiscence. Serious Adverse Events (SAEs): 2 deaths (1 cardiac, 1 ALS‐related), 2 pulmonary emboli, 2 pneumonia hospitalizations. No treatment‐related acceleration of ALS progression. Tolerability: 5/12 patients required dose reduction/discontinuation of immunosuppressants (tacrolimus/mycophenolate) due to GI toxicity (vomiting, diarrhea). Secondary Outcomes: Disease Progression: ALSFRS‐R/HHD/FVC: No worsening post‐intervention. EIM: Linear decline in active disease; stable in end‐stage patients. Patient 11: Anecdotal improvement in ALSFRS‐R/HHD (not statistically significant).	ALSFRS‐R/HHD/FVC: No acceleration of disease progression in any patient post‐intervention. Patient 11: Modest improvement in ALSFRS‐R and HHD (not statistically significant; trial not powered for efficacy). EIM: Consistent linear decline in EIM values (50 kHz phase) in patients with active disease; stable in end‐stage patients. No side‐to‐side differences between injected vs. non‐injected muscles. No Rejection/Infection: No HLA antibodies against donor cells; no opportunistic infections (e.g., CMV). Statistical Limitations: No *p*‐values/confidence intervals (phase I safety focus; efficacy not assessed).	Common: Transient radicular pain/sensory changes (surgery‐related). GI toxicity (diarrhea, vomiting) from immunosuppressants. Serious: Dural fistula (1), pulmonary emboli (2), deaths (2).
18	Mazzini et al. [34]	Italy	ALS	Phase I, Open‐Label, Non‐Randomized clinical trial. Single‐Arm	18 ALS patients—13 men and 5 women— 48 was the median age (range: 25–67).	Procedure: Intraspinal microinjections of human neural stem cells (hNSCs) into the gray matter of the lumbar or cervical spinal cord. Cohorts: Cohort 1: Non‐ambulatory patients (severe lower limb disability). Cohorts 2–3: Ambulatory patients (fair functional autonomy). Immunosuppression: Tacrolimus‐based regimen with routine monitoring.	No control group	Each location received 15 μL of injections of human neural stem cells at a concentration of 50,000 cells/μL, for a total of 750,000 cells/injection.	Cell Line: Forebrain‐derived hNSCs from two standardized, non‐immortalized human donors.	Cells injected into spinal cord gray matter.	Primary Outcomes: Safety: Immediate Risks: Allergic reactions (e.g., fever, rapid heart rate), respiratory failure, surgical complications (hematomas, infections), and new‐onset paralysis/sensory loss. Long‐Term Risks: Tumor formation, abnormal spinal reflexes (e.g., myoclonus), and persistent neurological deficits unrelated to ALS. Tolerability: Patient ability to withstand surgery, immunosuppression, and cell transplantation. Secondary Outcomes: Functional Decline: Measured via ALS‐FRS‐R (disease progression) and FVC (respiratory function). Quality of Life: Assessed using mood and life satisfaction questionnaires (Quality of Life Profile of Mood State, SEIQoL‐DW). Psychological/Behavioral Impact: Regular evaluations by clinical psychologists.	Disease Progression: Transient ALS‐FRS‐R Improvement: Slowed progression for 1–4 months post‐transplantation (*p* = 0.0136). Subjective Benefits: 5/18 patients reported temporary mobility improvements (2–6 months). 4/18 patients had short‐term upper limb functional gains. No Survival Benefit: 11 deaths due to ALS progression; autopsies showed no treatment‐related pathology. Respiratory Function (FVC): No significant changes. Quality of Life: Stable (mean SEIQoL score: 73%) despite disease progression. ALS‐FRS‐R: Statistically significant transient improvement (*p* = 0.0136); no confidence intervals reported. FVC: No significant differences.	Common: Mild postsurgical pain (resolved within 1–60 days); transient respiratory failure in 1 patient. Serious Events: 1 case of iatrogenic diabetes (insulin‐dependent). 2 cases of pneumonia (1 resolved with antibiotics, 1 requiring tracheotomy). 1 tacrolimus‐induced tremor (resolved after discontinuation).
19	Barczewska et al. [35]	Poland	ALS	Case–control study (a non‐randomized cohort study with historical controls)	Treatment group: 67 (37 male/30 female). Age: 57 (36–78) Reference group: 67 (37 male/30 female). Age: 58 (31–76)	Treatment Group: Received three intrathecal injections of Wharton’s jelly‐derived mesenchymal stem cells (WJ‐MSCs) at 2‐month intervals. Dosage: 30 million cells per injection.	Reference Group: Received standard ALS therapies from historical clinical trial data (no specific drugs detailed).	30 million cells per injection	Obtained from healthy newborns’ umbilical cords with post‐parental consent	Administered in 1 mL of 5% human albumin through lumbar puncture (L3/L4 level).	Primary: Survival: Median survival time and mortality risk reduction. Disease Progression: Rate of disability decline (ALSFRS‐R score). Secondary: Subgroup differences by baseline disability (FT9 stage). Predictive value of early treatment response.	Survival: 70% lower mortality risk in MSC group vs. controls (HR = 0.30; 95% CI: 0.16–0.59; *p* = 0.0004). Median survival doubled: 1183 days (MSC) vs. 640 days (controls; *p* = 0.002). Benefit consistent across disability stages (*p* = 0.03 for FT9 stages 1–2 and 3–5). Disease Progression: Slower decline during treatment vs. pre‐treatment (*p* = 0.008). Response Types: 31.3% (21/67) had slower progression. 49.3% (33/67) showed no change. 19.4% (13/67) had accelerated progression. Strong treatment effect (*p* = 0.0003) in regression analysis. Predictors: Early response after first MSC dose correlated with better long‐term outcomes (*p* = 0.0059).	No severe complications related to MSC therapy. Minor Issues: Post‐lumbar puncture syndrome (5 patients: transient headache/nausea). Low‐grade fever (2 patients). No malignancies, meningeal signs, or unexpected symptoms reported.
20	Martinez et al. [36]	Mexico	ALS	A prospective cohort study with a concurrent control group is known as a non‐randomized controlled trial.	10 Control group (5 male/5 female) Age: (31–70) 10 Treatment group (5 male/5 female) Age: (35–62)	Stem Cell Therapy: Bone Marrow Stimulation: Subcutaneous filgrastim (300 mg/day for 3 days). Leukapheresis: Peripheral blood mononuclear cells (PBMCs) collected. CD133+ Cell Isolation: Magnetic bead‐based separation (anti‐CD133 microbeads). Transplantation: CD133+ cells (2.5–7.5 × 10^5^ cells) suspended in 0.3 mL autologous cerebrospinal fluid (CSF) and injected into the frontal motor cortex via stereotactic surgery.	Control group; Rilizole (glutamate inhibitor) and antioxidants, no stem cell therapy.	2.5–7.5 × 10^5^ CD133+ cells per patient.	Autologous: Following filgrastim stimulation, CD133+ stem cells were extracted from the patient’s own peripheral blood.	Intracortical Implantation: Using a Hamilton syringe guided by stereotactic/neuronavigation, cells are injected bilaterally into the frontal motor cortex.	Primary: Survival time from diagnosis (tracheostomy/gastrostomy/death as endpoints). Secondary: Disease progression (ALSFRS‐R scores). Quality of life (Spitzer QOL scale). Respiratory function (FVC). Dose‐response relationship (cell count vs. outcomes).	Survival: Treatment Group: Median survival = 66 months (95% CI 17.53–114.47). Control Group: Median survival = 19 months (95% CI: 11.77–26.23). Significance: Log‐rank test, *p* = 0.0111 (treatment group survival doubled). Disease Progression: Treatment Group: ALSFRS‐R scores stabilized at 1 year (mean 24 vs. baseline 24.6). Control Group: Significant decline at 1 year (mean 15.7 vs. baseline 31.4; *p* < 0.001). Dose–Response: Higher CD133+ cell doses (>500,000 cells) correlated with better ALSFRS‐R scores (χ^2^ = 5.77, *p* = 0.0162). Respiratory Function: Treatment group maintained stable FVC (44.6% vs. control group’s decline to tracheostomy in 5 patients).	Treatment Group: Serious: 1 death due to myocardial infarction 10 days post‐surgery. Non‐Serious: Transient increase in white blood cells (mean 33,260 vs. baseline 6979) from G‐CSF; no infections or procedural complications. Control Group: Higher rates of tracheostomy/gastrostomy (5 patients) and mortality.
21	Prabhaka et al. [37]	India	ALS	Open‐label pilot study	10 patients (7 female/3 male) Mean Age: 49.1/After a year of follow‐up, 8 patients survived, Extended follow‐up saw 2 more deaths due to respiratory failure and 2 others due to massive aspiration.	Bone marrow aspiration: A disposable needle was used to take 30 to 100 mL of bone marrow aspirate from the iliac crest in a clean environment. To separate mononuclear cells, bone marrow aspirates were put on a Ficoll density (Ficoll–Pague) gradient and spun at 700 g for 25 min. We took the mononuclear cells out of the bone marrow aspirate. Processing cells: washing them with sterile phosphate‐buffered saline (PBS) and spinning them three times at 400 g for 5 min. The Trypan blue exclusion test checked for viability (>70% of cells were alive). Giemsa staining under a light microscope showed the shape of the cells. The number of cells was counted automatically by a hematology analyzer (Orion 30). Flow cytometry: Flow cytometry (FACS Aria) used CD‐34 and CD‐45 antibodies to find cells.	none	Bone Marrow Aspiration Volume: 30–100 mL (from iliac crest). There is no explicit mention of the precise dosage of MSCs given.	Autologous stem cells from bone marrow	Intrathecal instillation of stem cells was done within 4 h of bone marrow aspiration in all patients. Cells were injected into subarachnoid space through lumbar puncture (L2−3 or L3−4) by 22G disposable lumbar puncture needles observing all the aseptic precautions.	Primary outcome assessed: Composite ALSFRS‐R score at 90, 180, 270, and 365 days after stem cell transplantation Secondary outcomes assessed: ALSFRS‐R sub‐scores (bulbar, fine, gross motor) at 3 months (90 days). Time to 4‐point decline in composite ALSFRS‐R score. Median survival.	1. Baseline characteristics/2:40 Mean duration of illness before treatment: 18.3 months (±13.3 SD) Median duration of follow‐up: 19.5 months (range: 6.0–24.0) 2. ALSFRS‐R outcomes/2:45 Day 90 follow‐up: No significant difference in bulbar, fine motor, and gross motor sub‐scores of ALSFRS‐R (P not reported). ALSFRS‐R composite scores “significantly unchanged” from baseline (P not reported). Day 180 and 270 follow‐up: Statistically significant decline in total ALSFRS‐R score (P not reported) 1‐year follow‐up: Survivors showed no statistically significant decline in total ALSFRS‐R score (P not reported) Mean time to 4‐point ALSFRS‐R decline: 16.74 months (±2.78 SD) 3. Survival outcomes/3:10 At 1 year: 8/10 patients were alive Cause of death: Respiratory failure: 4 pts; Massive aspiration: 2 pts Median survival estimates: • From disease onset 33.0 (95 % CI: 23.4–42.6) • Post‐procedure: 18.0 (95 % CI: 8.7–27.3) 4. Comparison of baseline characteristics between survivors and non‐survivors/3:25 Median baseline ALSFRS‐R score: No significant difference between survivors and non‐survivors (*p* = 0.468) 5. Safety data/3:30 No significant adverse events of any kind were reported during follow‐up	No significant adverse events were observed.
22	Deda et al. [38]	Turkey	ALS	Interventional study Before and after, case series	13 participants:3 female,10 male Age: (34–71) Three patients passed away from MI and lung infections 1.5, 2, and 9 months following the procedure.	Revised Summary of Stem Cell Administration Protocols: 1. Intraspinal Cord Delivery Volume/Cells: 0.1 mL of cell suspension (cell quantity unspecified) administered to multiple spinal cord regions using a 21‐gauge needle. 2. Gel Foam Application Volume/Cells: 3 mL (≥10 million cells) infused into a gelatin‐based matrix positioned over the brainstem and lower cranial nerve regions. 3. Subarachnoid Space Injection Volume/Cells: 1.5 mL (~5 million cells) delivered directly to the operative site. 4. Intravenous Infusion Volume/Cells: 1.5 mL (~5 million cells) administered systemically. Total Cellular Dose: Minimum Estimate: ~20 million cells (10 million in Gel Foam + 5 million subarachnoid + 5 million intravenous). Note: The intraspinal injections (0.1 mL) lack quantified cell data, suggesting potential for higher totals. Treatment Timeline: Single Intervention: All cellular therapies were performed during a singular surgical session, with no documentation of subsequent doses or extended treatment regimens. Additional Specifications: Cell Source: Bone marrow‐derived mononuclear cells (MNCs), prepared through processing and cryopreservation prior to surgery. Post‐Operative Protocol: No evidence of additional stem cell therapies (e.g., booster doses) following the initial procedure.	None	Intraspinal Cord Injections: Volume/Cells: 0.1 mL (cell count unspecified) injected into multiple spinal cord areas via a 21‐gauge needle. Gel Foam Placement: Volume/Cells: 3 mL (≥10 million cells) injected into Gel Foam material placed on the brain stem and lower cranial nerve. Subarachnoid Space Injection: Volume/Cells: 1.5 mL (~5 million cells) injected at the surgical site. Intravenous Administration: Volume/Cells: 1.5 mL (~5 million cells) given intravenously. Total Administered Cells: Minimum: ~20 million cells (10 million in Gel Foam + 5 million subarachnoid + 5 million intravenous). Note: The 0.1 mL intraspinal injections are not quantified in cell count, so the total may exceed 20 million.	The iliac crest was used to aspirate bone marrow.	Intraspinal cord injections/subarachnoid space injection/intravenous administration	Primary outcomes: bulbar scores and Norris scales. Secondary outcomes: ENMG	After stem cell implantation, nine patients showed improvement, one remained stable, and three passed away from myocardial infarction and lung infection.	Some patients had improvements in speaking, swallowing, and breathing. One patient passed away from MI, while two more died from lung infections. No significant adverse effects mentioned in this study.
23	Mazzini et al. [39]	Italy	ALS	Phase I clinical trials	19 patients (11 male and eight‐ female)/Following surgery, eight patients passed away after an average life of 31.6 months.	Autologous mesenchymal stem cell (MSC) transplantation into ALS patients’ spinal cords was part of the study. MSCs were transplanted into the dorsal spinal cord at particular thoracic vertebrae (T7–T9 in the first experiment; T4–T6 in the second trial) after being separated from the patients’ bone marrow, grown in vitro, and suspended in autologous cerebrospinal fluid (CSF).	None	1a: 7 × 10^6^ 2a: 24 × 10^6^ 3a: 30 × 10^6^ 4a: 40 × 10^6^ 5a: 60 × 10^6^ 6a: 150 × 10^6^ 7a: 152 × 10^6^ 8a: 24 × 10^6^ 9a: 32 × 10^6^ 1b: 80 × 10^6^ 2b: 90 × 10^6^ 3b: 109 × 10^6^ 4b: 70 × 10^6^ 5b: 45 × 10^6^ 6b: 95 × 10^6^ 7b: 75 × 10^6^ 8b: 120 × 10^6^ 9b: 11 × 10^6^ 10b: 51 × 10^6^	Bone marrow	Surgical procedure	Primary Outcomes Safety and Feasibility Evaluation Survival rates Severe complications (e.g., surgical risks, tumor development, abnormal neural circuitry). Extended adverse effect monitoring (over 9 years), covering: Involuntary muscle spasms (myoclonus) Motor coordination deficits (ataxia) Pain syndromes Respiratory safety (prioritized by enrolling patients with stable baseline lung function). Secondary Outcomes Functional Assessments ALS Functional Rating Scale (ALS‐FRS): Evaluated daily functional capacity (e.g., swallowing, meal preparation, mobility). Second trial eligibility required thresholds: ≥3 for swallowing, ≥2 for food management, and ≥2 for walking. Forced Vital Capacity (FVC%): Tracked respiratory efficiency during follow‐ups. Psychological and Quality of Life (QoL) Metrics Profile of Mood State (POMS): Analyzed emotional fluctuations. Schedule for the Evaluation of Individual Quality of Life–Direct Weighting (SEIQoL‐DW): Personalized QoL assessment. Tailored Trial Feedback Survey: Captured patient insights on trial communication, procedural experiences, and post‐treatment effects. Included qualitative prompts about trial‐related perceptions and overall impressions. Neuroimaging Analysis MRI and Diffusion Tensor Imaging (DTI): Detected anatomical alterations in the central nervous system. Conducted at baseline, 15 days pre‐transplant, and post‐surgery intervals (15 days, 3–24 months, then annually/biennially). Follow‐Up Strategy Quarterly clinical reviews (ALS‐FRS, FVC%) continued until end‐of‐life. Remote monitoring (email/phone) for ALS‐FRS and psychological surveys in non‐ambulatory patients. Imaging schedules adjusted dynamically based on respiratory health. Data Handling All outcome data were archived in the National Institute of Health’s registry for gene and cell therapy trials.	Postoperative Survival Average survival: 31.6 months (±21 SD; range: 9–74 months) after surgery. Mortality causes: 6/8 patients: Disease progression (ALS). 1/8: Stroke (hemorrhagic). 1/8: Pulmonary embolism. Safety: No fatalities attributed to MSC transplantation. 2. Functional Stability and Decline Functional plateaus: 6 patients (2a, 4a, 6a, 9a, 1b, 2b) showed ≤10% fluctuation in ALS‐FRS and FVC% scores. Stability duration: Longest: 74 months (patients 7a, 9a). Shorter: 30 months (patients 2a, 1b, 2b). Decline: Functional deterioration followed stable periods. 3. Psychological and Quality of Life (QoL) Insights SEIQoL‐DW scores (Mean ± SD): Baseline: 56.6 ± 17.4. 1‐year post‐transplant: 57.0 ± 20.8. Final assessment: 57.1 ± 18.7. QoL priorities: Health, family, and friendships (all patients); career/studies (6/11 patients). Mood assessment: Only 1/11 patients reported significant mood disturbances (POMS). Satisfaction: 10/11 participants viewed trial involvement positively. 4. Radiological Findings Safety: No abnormalities: Absence of tumors, syringomyelia, or corticospinal tract alterations (MRI/DTI). Spinal cord changes: Temporary enlargement: Anteroposterior (AP) and right‐left (RL) spinal cord diameters at injection site post‐surgery, normalizing over time.	Neither surgery nor MSC transplantation were linked to any fatalities. MRI/DTI revealed no signs of tumors, syringomyelia, pseudomeningocoele, or anomalies of the corticospinal tract. After treatment, no further mental health assistance was needed. No signs of iatrogenic injury, cancer, or abnormal neural connections.
24	Rushkevich et al. [40]	Belarus	ALS	Quasi	10 patients in the main group had autologous MSC transplantation, while 15 patients in the control group received standard symptomatic treatment. Ages of the main group (54.5) and comparison group (55)	Stem Cell Therapy: IV Injection: 0.5–1.5 × 10^6^ cells/kg (42–102 × 10^6^ total cells) via catheter. Intra lumbar Injection: 5.0–9.7 × 10^6^ cells via lumbar puncture (L3–L4). Frequency: 6 patients (1 course); 4 patients (2 courses, 5–7‐month interval). Control Group: Standard drugs (neurometabolites, anticonvulsants, antidepressants).	Control	Main Group: IV Injection: 0.5–1.5 × 10^6^ cells/kg (42–102 × 10^6^ total cells) via catheter Intra lumbar Injection: 5.0–9.7 × 10^6^ cells via lumbar puncture (L3–L4).	Bone marrow mesenchymal stem cells (MSC)	Main Group: IV Injection: Infused via 18G catheter in saline +5% autologous serum. Intra lumbar Injection: Injected via lumbar puncture (L3–L4) into the subarachnoid space.	Primary: Functional decline (ALSFRS‐R, Karnofsky Scale). Muscle strength (Manual Muscle Scale). Secondary: Neurophysiological changes (ENMG). MSC phenotype and neural differentiation markers (NSE, NESTIN, MAP2 via RT‐PCR).	Neurogenic Markers: NSE: Increased expression post‐induction (*p* = 0.0077). NESTIN: Significant upregulation (*p* = 0.0012). MAP2: No change (*p* = 0.36). Functional Scores: ALSFRS‐R: MSC group median 34 vs. control 16 (*p* < 0.001). Karnofsky: MSC group 70% vs. control 20% (*p* < 0.001). Disease Progression: Slower in MSC group (e.g., ALSFRS‐R decline 0.38 vs. 1.47, *p* = 0.003). Bulbar Syndrome: Delayed onset in MSC group (e.g., *p* = 0.008 at 9 months). Mortality: Lower in MSC group (20% vs. control 46.7%).	Minor, transient issues: Fever (1 patient, resolved in 2 h). Post‐lumbar puncture headaches (2 patients, managed conservatively). No severe complications (e.g., infections, structural CNS damage).
25	Mazzini et al. [41]	Italy	ALS	Before and after	9 patients (5 Females and 4 Males). 4 patients died.	Autologous MSCs injected intra spinally at the thoracic level. There were 57 × 10^6^ cells implanted on average (range: 7.0 × 10^6^–152 × 10^6^). Under microscopical vision, a laminectomy was carried out, and the dura was opened along the median line. Under microscopical vision, a laminectomy was carried out, and the dura was opened along the median line.	None	There were 57 × 10^6^ cells implanted on average (range: 7.0 × 10^6^–152 × 10^6^).	The iliac crest was the source of the bone marrow.	The cells were injected 1 mm apart in the most central section of the spinal cord by means of the ago cannula (18 ga) syringe previously mounted in an injection system with a micrometric pump injector supported by a table‐ fixed arm.	Primary: ALS‐FRS, Norris score, FVC, Bulbar function. Secondary: Psychological well‐being (POEMS, SEIQoL‐DW), MRI/SEP stability (no structural or neurophysiological abnormalities).	MSC Characteristics: Patient‐derived mesenchymal stem cells (MSCs) showed no functional or genetic differences compared to healthy donor MSCs. Clinical Outcomes: Functional Decline: 4/9 patients experienced a transient slowdown in Forced Vital Capacity (FVC) decline; 1 patient maintained normal respiratory function for 51 months post‐transplant despite disease progression. Survival: No correlation between survival and baseline clinical features. Three deaths occurred due to ALS progression (respiratory failure at 9, 24, and 44 months).	Severe Events: None linked to MSC therapy (e.g., no persistent neurological deficits or MRI abnormalities). Minor Events: Temporary intercostal pain (4 patients) and leg sensory dysesthesia (6 patients), resolving within days/weeks.
26	Blanquer et al. [42]	Spain	ALS	Open single‐arm Phase I trial	11 patients (5 males, 6 females); Median age: 46 years; Median disease duration: 21 months	Intraspinal infusion of autologous bone marrow mononuclear cells (BMNCs) into the posterior spinal cord funiculus	None	462 × 10^6^ BMNCs infused	Autologous bone marrow	Intraspinal (posterior spinal cord funiculus)	Primary: Safety (serious adverse events); Secondary: FVC, ALS‐FRS, Norris, MRC scales	No severe transplant‐related adverse events; 43 non‐severe events (51% resolved in <2 weeks); No significant decline in FVC, ALS‐FRS, Norris, or MRC scales observed	43 non‐severe adverse events (e.g., constipation, surgical wound pain); 6 severe adverse events unrelated to the procedure; No deterioration in neurological scales
27	Karussis et al. [43]	Greece	Multiple sclerosis (MS) and ALS	Phase 1/2 open‐safety clinical trial	34 patients (15 MS, 19 ALS); MS mean EDSS: 6.7 [1.0], ALS mean ALSFRS: 20.8 [8.0]	Intrathecal and intravenous administration of autologous mesenchymal stem cells (MSCs)	None	63.2 × 10^6^ MSCs injected intrathecally; 24.5 × 10^6^ MSCs intravenously	Autologous bone marrow	Intrathecal and intravenous	Primary: Safety (adverse events); Secondary: EDSS, ALSFRS, MRI, immunological effects	21 patients had mild injection‐related adverse effects; EDSS improved from 6.7 to 5.9; ALSFRS stable	21 mild adverse events (e.g., fever, headache); No major adverse effects reported
28	Riley et al. [44]	USA	ALS	Phase I trial	12 patients; Age: Not specified; Gender: Not specified	Intraspinal infusion of neural stem cells; 5 injections (10 mL each) at 4 mm intervals	None	100,000 neural stem cells per injection	Fetal spinal cord‐derived	Intraspinal (upper lumbar segments)	Primary: Safety (serious adverse events); Secondary: Neurological function, urinary retention, somatosensory‐evoked potentials	No major motor function decrement; 1 transient intraoperative event; 2 readmissions for complications; 2 deaths unrelated to the procedure	43 non‐severe adverse events (e.g., transient fever, headache); 1 episode of urinary retention; 2 patients required reoperation for complications
29	Riley et al. [45]	USA	ALS	Phase I trial	15 patients; Age: 35–56; 4 males, 2 females	Microinjection of NSI‐566RSC; 5 injections (10 mL each) at 4 mm intervals	None	1.0 × 10^4^ cells/mL per injection	Human fetal spinal cord‐derived	Intraspinal (cervical and thoracolumbar)	Primary: Safety (adverse events); Secondary: Neurological function, vital capacity, ALSFRS‐R scores	No significant motor function decrement; 1 patient developed kyphotic deformity; 1 reoperation for wound dehiscence; 1 reintubation due to bulbar ALS	2 patients required reoperation; 1 patient developed superficial wound infection; no major adverse events related to the procedure
30	Tarella et al. [46]	Italy	ALS	Multicenter prospective phase II trial	24 patients Age: Median 57 years (range 40–64) Gender: 12 male, 12 female Diagnosis: Definite (16), Probable (6), and Probable with lab support (2)	SC Mobilization: G‐CSF (5 μg/kg every 12 h × 4 days, repeated every 3 months × 4 courses) Mannitol: 125 mL 18% solution IV × 5 days to enhance BBB permeability	None	Median peak CD34+ cells/μL: 57 (1st course), 57 (2nd), 46 (3rd), 41 (4th)	Peripheral blood mobilized by G‐CSF	G‐CSF: Subcutaneous Mannitol: Intravenous	Primary: Safety/tolerability of G‐CSF Secondary: ALS progression (ALS‐FRS‐*r* score, respiratory capacity) CD34+/progenitor cell mobilization	Safety: No ALS worsening (*p*‐value not reported) CD34+ markers: ↑CD133 (*p* < 0.0001), ↓CD184 (*p* < 0.05) Survival: 20/24 alive at 12 months	Common: Bone pain, flu‐like symptoms, and headache Severe: Transient hyperprolactinemia (1) and DVT (1) Other: Leukocytosis >75,000/μL (1)
31	Sharma et al. [47]	India	ALS	Retrospective controlled cohort study	57 patients (37 intervention, 20 control); Mean age: 48.89 (10.79) for intervention, 53.11 (11.49) for control; 15 females (40.54%), 22 males (59.46%) in intervention; 8 females (40.00%), 12 males (60.00%) in control	Autologous bone marrow mononuclear cell transplantation; Standard rehabilitation; Riluzole; Lithium (300 mg twice daily for 6 weeks)	20 patients who did not receive cell transplantation	The average cell count was 80.5 × 106 with 96.7 % average cell viability.	Autologous bone marrow	Intrathecal and intramuscular	Primary: Survival duration since onset of disease; Secondary: Age at onset, Lithium effect, Bulbar vs. Limb onset	Mean survival duration: 87.76 (10.45) months (intervention) vs. 57.38 (5.31) months (control); *p* = 0.039 for age < 50 years; *p* = 0.121 for lithium effect	Minor side effects: nausea, spinal headache, vomiting, pain at injection site, parasthesia in lower limbs

Abbreviations: 6MWT, 6‐Minute Walk Test; AALS, Appel ALS scores; AE, adverse event; AF, atrial fibrillation; ALSFRS‐R, Amyotrophic Lateral Sclerosis Functional Rating Scale; ASCT, autologous stem cell transplantation; ASIA Scale, American Spinal Injury Association Impairment Scale; Auto‐PBSCT, autologous peripheral blood stem cell transplantation; BMD, Becker muscular dystrophy; CAP‐1002, clinical trial of allogeneic cardiosphere‐derived cells; CD, cluster of differentiation; CDC, cardiosphere‐derived cell; CDCs, cardiosphere‐derived cells; CHIT‐1, chitinase‐1; CK‐MB, creatine kinase‐MB; CMAP, compound muscle action potential; CPK, creatine phosphokinase; CRH/VGPRH, complete/very good partial remission; CSF, cerebrospinal fluid; CT, computed tomography scan; cTn, cardiac troponin; CXR, chest radiograph (chest X‐ray); DLCO, diffusing capacity of the lungs for carbon monoxide; DMD, Duchenne muscular dystrophy; DSPN, diabetic sensorimotor polyneuropathy; DSA, donor‐specific antibody; ECOG, Eastern Cooperative Oncology Group; EEG, electroencephalography; EIM, electrical impedance myography; ELISA, enzyme‐linked immunosorbent assay; EMG, electromyography; ENMG, electroneuromyography; FACS, fluorescence‐activated cell sorting; FEV1, forced expiratory volume in 1 second; FVC, forced vital capacity; G‐CSF, granulocyte colony‐stimulating factor; HHD, hand‐held dynamometry; HLA, human leukocyte antigen; HOPE, Halt Cardiomyopathy Progression; HSSCs, hematopoietic stem and progenitor cells; hUC‐MSCs, human umbilical cord mesenchymal stem cells; ICU, intensive care unit; IL, interleukin; IMWG criteria, International Myeloma Working Group criteria; IT, intrathecal; IV, intravenous; LGE, late gadolinium enhancement; LGMD, limb‐girdle muscular dystrophy; LV, left ventricular; LVAD, left ventricular assist device; LVEDV, left ventricular end‐diastolic volume; LVEF, left ventricular ejection fraction; LVESV, left ventricular end‐systolic volume; MACE, major adverse cardiac events; MAP2, microtubule‐associated protein 2; MCB, master cell bank; MCP‐1, monocyte chemoattractant protein‐1; MCV, motor conduction velocities; MEP, maximal expiratory pressure; MFI, mean fluorescence intensity; MIP, maximal inspiratory pressure; mITT, modified intent‐to‐treat; mRS, modified Rankin Scale; MSCs, mesenchymal stem cells; MUNIX, motor unit number index; NCS, nerve conduction study; NCV, nerve conduction velocity; NESTIN, neuro‐intermediate filament protein; NfL, serum neurofilament light chain; NIS, Neuropathy Impairment Score; NSAIDs, nonsteroidal anti‐inflammatory drugs; NSE, neuron‐specific enolase; NTFs, neurotrophic factors; ONLS, overall neuropathy limitations scale; PBSCT, peripheral blood stem cell transplantation; PedsQL, Pediatric Quality of Life; PODCI, Pediatric Outcomes Data Collection Instrument; POEMS, polyneuropathy, organomegaly, endocrinopathy, M‐protein, and skin changes syndrome; PUL, performance of the upper limb; QoL, quality of life; RT‐PCR, reverse transcription polymerase chain reaction; RV, residual volume; SAE, serious adverse event; SDF‐1α, stromal cell‐derived factor‐1α; SEIQoL‐DW, Schedule for the Evaluation of Individual Quality of Life–Direct Weighting; SSEP, somatosensory evoked potentials; SVC, slow vital capacity; TEAE, treatment‐emergent adverse event; TGF, transforming growth factor; TIMI, thrombolysis in myocardial infarction; TLC, total lung capacity; TRUMP, Transplant Registry Unified Management Program; UTI, urinary tract infection; VEGF, vascular endothelial growth factor.

### 2.5. Methodological Quality Assessment

Methodological rigor and risk of bias were evaluated independently by two researchers (Soraya Babaie and Mahdieh Pourasghari) in alignment with the Cochrane Handbook for Systematic Reviews of Interventions [48].

For RCTs, bias domains included selection bias (randomization sequence generation, allocation concealment), performance bias (blinding of participants/personnel), detection bias (blinding of outcome assessors), attrition bias (completeness of outcome data), and reporting bias (selective outcome reporting). The risk of bias was visualized using Review Manager 5.3 (RevMan, Cochrane Collaboration). For non‐randomized studies, the ROBINS‐I (Risk Of Bias In Non‐randomized Studies of Interventions) tool was applied [49]. Discrepancies in assessments were resolved through consensus or adjudication by a third reviewer (Bina Eftekharsadat).

### 2.6. Data Synthesis and Analysis

Qualitative and quantitative syntheses were employed: qualitative synthesis for single‐arm before‐after studies and RCTs without similar outcomes or sufficient data. Meta‐analysis was done for RCTs with similar outcomes (ALSFRS) using RevMan 5.3. Moreover, pooling data was done for the single‐arm studies which had been conducted in two phases, included pre‐intervention control phase and interventional phase (MSCs injections). In this part of analysis, eligible study outcomes consisted of ALSFRS and FVC. Continuous outcomes were analyzed via weighted mean difference (WMD) with 95% confidence intervals (CI). Pre‐post intervention or control changes were calculated as: The mean difference in each group was computed by subtracting the post‐intervention mean from the pre‐intervention mean. The standard deviation was calculated using the formula below:

SD=SDb2+SDf2−2×r×SDb×SDf.

In this formula, SD_*b*_ represents the standard deviation at study beginning, SD_*f*_ denotes the standard deviation at follow‐up, and *r* indicates the correlation between the baseline and follow‐up values [49, 50]. For studies reporting median and interquartile range (IQR), mean, and SD were estimated using established conversion methods. The Universal Desktop Ruler 3.8 software was used to extract values from graphical representations. Heterogeneity was quantified via the *I* ^2^ statistic and Cochran’s Q‐test (*χ* ^2^). A random‐effects model was prioritized for analyses with *I* ^2^ >50% rather than a fixed effect, indicative of substantial heterogeneity. Subgroup analysis was done according to the treatment dose (≥1 × 10^6^ cells or <1 × 10^6^ cells) for RCTs. In addition, for single‐arm two‐phase trials, sub‐group analyses were done according to the treatment dose (low dose: 1–10 × 10^6^ cells, medium dose: 1–10 × 10^7^ cells, and high dose: 1–10 × 10^8^ cells). Furthermore, for two phases of single‐arm studies, sensitivity analyses were done based on the route of injections by remaining only IT injections as well as by removing high‐dose injections for FVC outcome.

## 3. Results

### 3.1. Literature Search and Study Selection

**Figure 1:**
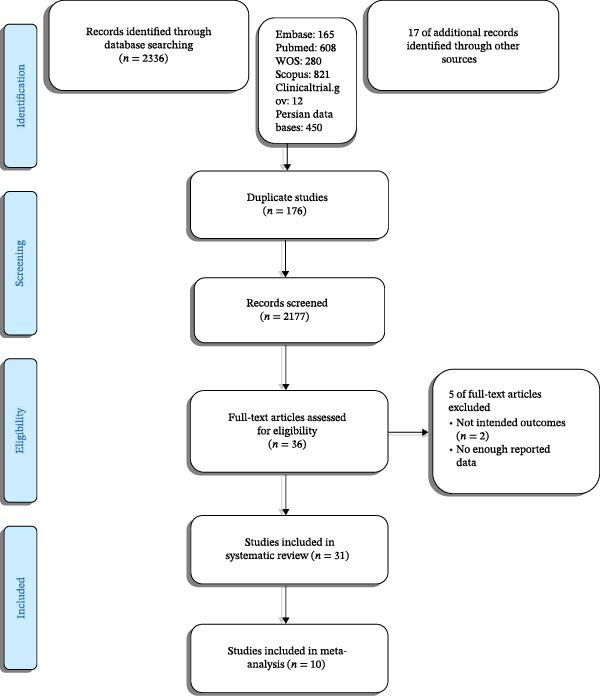
Preferred reporting items for systematic reviews and meta‐analyses (PRISMA) flowchart.

The article selection process, including exclusion rationales, is detailed in the PRISMA flow diagram (1). Initial database queries and supplementary sources yielded 2353 records. After removing 176 duplicates, 2177 studies underwent the eligibility screening. Full‐text screening identified 36 candidate studies, with 31 meeting inclusion criteria after full‐text review. Among these, 10 (3 controlled trials + 7 single‐arm studies) were eligible for quantitative synthesis (Figure 1).

### 3.2. Study Characteristics

The 31 included studies spanned publication years 2009–2023. Seven controlled trials [24, 30, 31, 35, 36, 40, 47] involved 370 participants, with 204 allocated to interventions and 166 to control conditions. Among these, 3 studies with control groups were included in the meta‐analysis [31, 36, 40]. An additional 460 participants were analyzed in 24 non‐controlled pre–post studies [17–23, 25–29, 32–34, 37–39, 41–46]. All studies were published in English and originated from Korea [17, 31], Iran [18, 26], Israel [19, 21, 23, 29], Austria [20], Czech Republic [22], Spain [24, 42], Italy [25, 34, 39, 41, 46], Poland [27, 35], USA [28, 30, 32, 33, 44, 45], Mexico [36], India [37, 47], Turkey [38], Belarus [40], and Greece [43]. The oldest mean age reported is from Gotkine et al. [19], with a mean age of 63 years in the low dose group. The youngest mean age reported is from Oh et al. [17], with a mean age of 45.7 years for the 7 patients. Age distributions varied across studies, where the minimum age of participants across studies was 20 years (Ruiz‐Navarro and Kobinia [20] and Petrou et al. [21]), and the maximum age was 80 years (Ruiz‐Navarro and Kobinia [20]).

### 3.3. Types of Stem Cells Utilized in Studies

In the analyzed studies, various types of stem cells were utilized, primarily focusing on autologous sources. The most commonly used stem cells were bone marrow‐derived MSC (BM‐MSCs), which were employed in multiple studies, including those by Oh et al. [17], Nabavi et al. [18], Ruiz‐Navarro and Kobinia [20], Sykova et al. [22], Shamsaei et al. [26], Petrou et al. [21, 23], Geijo‐Barrientos et al. [24], Berry et al. [30], Oh et al. [31], Prabhakar et al. [37], Deda et al. [38], Mazzini et al. [39], Rushkevich et al. [40], Mazzini et al. [41], Blanquer et al. [42], Karussis et al. [43], and Sharma et al. [47]. Other sources included human embryonic stem cells (Gotkine et al. [19]), fetal human neural stem cells (Mazzini et al. [25], Glass et al. [29], Feldman et al. [32], Glass et al. [33], Mazzini et al. [34], and Riley et al. [44]), and Wharton’s Jelly‐derived stem cells (Barczewska et al. [27]).

### 3.4. Variability in Stem Cell Dosing Regimens

The dosing regimens varied significantly across studies. The minimum doses of stem cells reported are 0.27 × 10^6^ cells/kg, as noted by Barczewska et al. [27], administered via IT injection, and 2.5–7.5 × 10^5^ CD133+ cells per patient, as noted by Martinez et al. [36]. In contrast, the maximum doses are 462 × 10^6^ BMNCs per patient from Blanquer et al. [42] and 250 × 10^6^ cells from Gotkine et al. [19], also delivered through IT injection.

The reviewed studies utilized five distinct administration routes for stem cell delivery in ALS, with IT being the most common route, employed in 18 studies: Oh et al. [17, 31], Nabavi et al. [18], Gotkine et al. [19], Ruiz‐Navarro and Kobinia [20], Petrou et al. [21], Syková et al. [22], Petrou et al. [23], Shamsaei et al. [26], Barczewska et al. [27, 35], Staff et al. [28], Berry et al. [30], Prabhakar et al. [37], Deda et al. [38], Rushkevich et al. [40], Karussis et al. [43], and Sharma et al. [47]. Other routes included IV in four studies (e.g., Nabavi et al. [18], Karussis et al. [43], Ruiz‐Navarro and Kobinia [20], Rushkevich et al. [40]), IM in five studies (e.g., Geijo‐Barrientos et al. [24], Berry et al. [30], Ruiz‐Navarro and Kobinia [20], Petrou et al. [21], and Sharma et al. [47]), intraspinal injections (Mazzini et al. [25, 34, 41], Glass et al. [29, 33], Feldman et al. [32], Blanquer et al. [42], Riley et al. [44, 45], and Deda et al. [38]), and subcutaneous in Tarella et al. [46]. Notably, nine studies combined multiple routes to target systemic and localized disease mechanisms: Nabavi et al. [18] (IT + IV), Ruiz‐Navarro and Kobinia [20] (IT + IM + IV), Petrou et al. [21] (IT + IM), Berry et al. [30] (IT + IM), Deda et al. [38] (intraspinal + subarachnoid + IV), Rushkevich et al. [40] (IV + IT), Karussis et al. [43] (IT + IV), Tarella et al. [46] (subcutaneous + IV), and Sharma et al. [47] (IT + IM).

### 3.5. Repeated Dosing Schedules With Defined Intervals

The analysis of the referenced clinical studies reveals two distinct methodological approaches to therapeutic administration. While the majority of investigations employed a single‐administration protocol, a significant subset utilized multiple injection regimens with varying temporal intervals. These repeated dosing protocols demonstrated considerable diversity in their design: Oh et al. [17, 31] implemented dual‐injection schedules separated by 26‐day intervals; Petrou et al. [23] administered between one and four IT injections at 3–6‐month intervals, while Barczewska et al. [27, 35] consistently applied triple‐injection protocols with 2‐month inter‐dose periods. Additional multi‐dose strategies included bi‐monthly injections in Staff et al. [28], quarterly treatment courses in Tarella et al. [46], and extended‐interval secondary courses in Rushkevich et al. [40] administered after 5–7‐month intervals. This heterogeneity in dosing schedules reflects ongoing investigations into optimal administration protocols for cell therapies.

### 3.6. Risk of Bias in Included Studies

**Figure 2:**
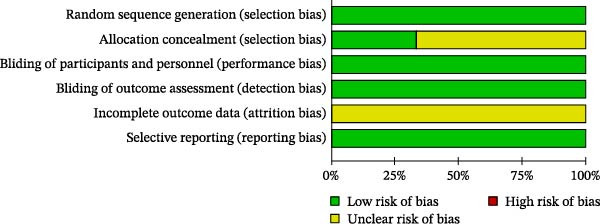
Risk of bias graph: review authors’ judgements about each risk of bias item presented as percentages across all included studies.

**Figure 3:**
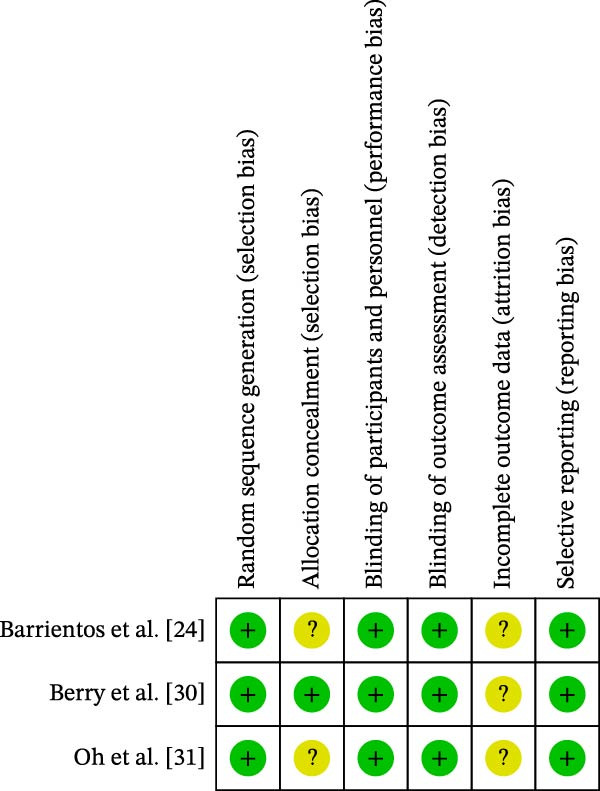
Risk of bias summary: review authors’ judgements about each risk of bias item for each included study.

Methodological quality assessments, based on independent reviewers’ judgments of bias domains for each study, are summarized in Table 2 and Figures 2 and 3. Most of the trials included displayed a low risk and unclear of bias, respectively. The lowest risk of bias (100% low risk) was related to random sequence generation (selection bias), blinding of participants and personnel (performance bias), blinding of outcome assessment (detection bias), and selective reporting (reporting bias). The highest risk of unclear bias (100%) was related to incomplete outcome data (attrition bias).

**Table 2:** Risk of bias in quasi‐experimental studies based on ROBINS‐I.

Studies	Bias due to confounding	Bias in selection of participants into the study	Bias in classification of interventions	Bias due to deviations from intended interventions	Bias due to missing data	Bias in measurement of outcomes	Bias in selection of the reported result	Overall
Barczewska et al. [35]	Serious risk	Low risk	Low risk	Low risk	Moderate risk	Low risk	Low risk	Moderate risk
Martinez et al. [36]	Serious risk	Moderate risk	Low risk	Low risk	Low risk	Low risk	Low risk	Moderate risk
Prabhakar et al. [37]	Serious risk	Moderate risk	Low risk	Low risk	Low risk	Low risk	Low risk	Moderate risk
Deda et al. [38]	Serious risk	Moderate risk	Low risk	Low risk	Low risk	Low risk	Low risk	Moderate risk
Mazzini et al. [39]	Serious risk	Moderate risk	Low risk	Low risk	Low risk	Low risk	Moderate risk	Moderate risk
Sharma et al. [47]	Serious risk	Moderate risk	Low risk	Low risk	Low risk	Low risk	Low risk	Moderate risk
Mazzini et al. [34]	Serious risk	Moderate risk	Low risk	Low risk	Serious risk	Low risk	Low risk	Serious risk
Glass et al. [29]	Serious risk	Moderate risk	Low risk	Moderate risk	Low risk	Low risk	Low risk	Moderate risk
Feldman et al. [32]	Serious risk	Moderate risk	Low risk	Low risk	Moderate risk	Low risk	Moderate risk	Moderate risk
Mazzini et al. [41]	Serious risk	Moderate risk	Low risk	Low risk	Low risk	Low risk	Moderate risk	Moderate risk
Rushkevich et al. [40]	Serious risk	Moderate risk	Low risk	Low risk	Low risk	Low risk	Serious risk	Moderate risk
Oh et al. [17]	Serious risk	Moderate risk	Low risk	Low risk	Low risk	Low risk	Low risk	Moderate risk
Staff et al. [28]	Serious risk	Moderate risk	Low risk	Moderate risk	Moderate risk	Low risk	Low risk	Moderate risk
Barczewska et al. [27]	Serious risk	Moderate risk	Low risk	Low risk	Low risk	Low risk	Low risk	Moderate risk
Shamsaei et al. [26]	Serious risk	Moderate risk	Low risk	Low risk	Moderate risk	Low risk	Moderate risk	Moderate risk
Mazzini et al. [25]	Serious risk	Low risk	Low risk	Low risk	Moderate risk	Low risk	Low risk	Serious risk
Petrou et al. [23]	Serious risk	Moderate risk	Low risk	Serious risk	Serious risk	Low risk	Moderate risk	Serious risk
Syková et al. [22]	Serious risk	Moderate risk	Low risk	Low risk	Low risk	Low risk	Moderate risk	Serious risk
Petrou et al. [21]	Serious risk	Serious risk	Moderate risk	Serious risk	Serious risk	Low risk	Low risk	Moderate risk
Ruiz‐Navarro and Kobinia [20]	Serious risk	Moderate risk	Low risk	Moderate risk	No information	Low risk	Moderate risk	Moderate risk
Gotkine et al. [19]	Serious risk	Moderate risk	Low risk	Moderate risk	Moderate risk	Low risk	Moderate risk	Moderate risk
Nabavi et al. [18]	Serious risk	Moderate risk	Low risk	Low risk	Low risk	Low risk	Low risk	Moderate risk
Oh et al. [17]	Serious risk	Moderate risk	Low risk	Low risk	Low risk	Low risk	Low risk	Moderate risk
Blanquer et al. [42]	Serious	Serious	Low	Low	Low	Moderate risk	No information	Serious
Karussis et al. [43]	Serious	Moderate risk	Low	Low	No information	Moderate risk	No information	Serious
Riley et al. [44]	Serious	No information	Low	Low	No information	Moderate risk	No information	Serious
Riley et al. [45]	Serious	No information	Low	Low	No information	Moderate risk	No information	Serious
Tarella et al. [46]	Serious	Moderate risk	Low	Low	No information	Moderate risk	No information	Serious

Based on a ROBINS‐I assessment of 28 studies (Table 2), the most significant and universal methodological limitation was a serious risk of bias due to confounding as all studies lacked control groups. In contrast, risks were generally low for the classification of interventions and deviations from intended protocols and predominantly low to moderate for participant selection, missing data, outcome measurement, and selection of reported results.

### 3.7. Systematic Review of the Included 31 Studies

#### 3.7.1. ALSFRS and FVC

Among the 31 included studies, most studies evaluated functional outcomes using the ALSFRS. Overall, the evidence does not consistently demonstrate sustained clinical efficacy, and the observed pattern is more compatible with a temporary reduction in the rate of functional decline rather than disease reversal or durable disease modification.

The findings were grouped into three categories. First, several studies reported a statistically significant slowing of the ALSFRS decline after stem cell administration. Notable examples include Oh et al. [17], showed a reduction in the monthly ALSFRS decline from −1.38 to −0.04 points following IT MSC treatment (*p*  < 0.001); Oh et al. [31] in other study, demonstrated significantly less functional deterioration than controls at 4 and 6 months; Petrou et al. [23], reported a reduction in disease progression slope after repeated MSC administration (*p* = 0.0038); Syková et al. [22], demonstrated a significant slowdown at 3 months (*p*  < 0.02) and 6 months (*p*  < 0.05); and Shamsaei et al. [26], found a significant reduction in ALSFRS decline after treatment. Barczewska et al. [35] also reported slower disability progression during treatment. Second, some studies showed limited, subgroup‐specific, or short‐lived signals of efficacy rather than a robust overall benefit. Berry et al. [30] did not demonstrate a significant overall improvement but reported favorable responder outcomes in rapidly progressing patients treated with MSC‐NTF. Gotkine et al. [19] observed attenuation of ALSFRS decline during the first 3 months, although this effect was not maintained at a later follow‐up. Petrou et al. [21] reported improvement trends that approached but did not reach conventional statistical significance (*p* = 0.052), while Ruiz‐Navarro and Kobinia [20] described temporary stabilization in some patients but did not prevent overall ALSFRS decline. Third, multiple studies did not show meaningful improvement in ALSFRS outcomes and primarily supported treatment safety rather than efficacy. Studies by Nabavi et al. [18], Staff et al. [28], Glass et al. [29, 33], Mazzini et al. [25], Blanquer et al. [42], and Karussis et al. [43] either reported continued disease progression, no statistically significant functional benefit, or only absence of accelerated decline after treatment. A recurring observation across studies was that any apparent functional benefit tended to emerge early, often within 1–3 months, reach its maximal effect around 3–6 months, and then diminish over time.

Regarding FVC, evidence for respiratory benefit was weaker and less consistent than for ALSFRS Most studies did not demonstrate meaningful improvement in pulmonary function, although some reported temporary slowing of FVC decline. Several studies suggested potential respiratory benefit. Shamsaei et al. [26] reported a significant reduction in FVC decline from −12.6 ± 5.22% per 10 months before treatment to −4.8 ± 14.72% after treatment (*p*  < 0.001). Petrou et al. [21] observed improvement in FVC trajectory, changing from −2.6% per month to +0.86% per month in the combined IT and intramuscular (IM) treatment cohort (*p* = 0.036). Petrou et al. [23] in another study also reported slower respiratory deterioration after repeated MSC injections, although this effect did not reach statistical significance. Other studies reported stabilization rather than an improvement. Syková et al. [22] found that 80% of patients maintained FVC ≥70% at 9 months and 60% at 12 months, suggesting partial preservation of respiratory function. Martinez et al. [36] reported relatively stable respiratory capacity in treated patients compared with progressive decline in controls. Mazzini et al. [39, 41] described periods of temporary respiratory stability or slower decline in selected patients without durable benefit. In contrast, several studies found no measurable effect on FVC outcomes. Oh et al. [17] reported no significant change in FVC despite improvements in ALSFRS (*p* = 0.64). Nabavi et al. [18] similarly found no treatment‐related impact on respiratory decline (*p* = 0.731), and Oh et al. [31] showed no significant between‐group difference in FVC despite favorable functional outcomes. Overall, current evidence suggests that stem cell therapy may transiently attenuate respiratory decline in some patients, but a consistent or durable preservation of pulmonary function has not been demonstrated.

#### 3.7.2. Impact of Stem Cells Administration on Survival

In the current review, the five controlled studies investigated the effect of cell therapy on survival. In the study by Oh et al. [31], no significant difference in survival was observed between the MSC group and the control group (mean survival: 55 vs. 48 months; *p* = 0.487), although younger individuals showed a trend toward longer survival (65 months vs. 48 months; *p* = 0.10). The Mazzini et al. [34] study reported no survival benefit, with 11 deaths attributed to ALS progression. In Barczewska et al. [35], the MSC group exhibited a 70% lower mortality risk compared to controls (HR = 0.30; 95% CI: 0.16–0.59; *p* = 0.0004), and median survival doubled (1183 vs. 640 days; *p* = 0.002). This benefit was consistent across different disability stages (*p* = 0.03 for FT9 stages 1–2 and 3–5). In the study by Martinez et al. [36], the median survival in the treatment group was 66 months, while the control group had a median survival of 19 months (*p* = 0.0111). Additionally, Sharma et al. [47] reported a mean survival duration of 87.76 months in the intervention group compared to 57.38 months in the control group (*p* = 0.03). Overall, the results indicate a positive potential for cell therapy in improving survival in some studies, while others reported varying outcomes.

#### 3.7.3. Adverse Events by Dose and Route of Stem Cells Administration

In studies utilizing doses of stem cells less than 1 million cells, the reported adverse events were generally mild. For instance, Feldman et al. [32] administered 100,000 neural stem cells per injection, leading to mild side effects such as headaches and nausea. Mazzini et al. [34] noted that 750,000 cells per injection resulted in mild adverse events, primarily headaches and transient pain at the injection site. Martinez et al. [36] reported fatigue and mild fever in patients receiving doses of 2.5–7.5 × 10^5^ CD133+ cells. Additionally, Riley et al. [44, 45] documented mild adverse events such as fatigue and headaches in patients receiving low doses. The routes of administration for these studies varied, with some utilizing IV and others using IMor IT methods. Overall, most adverse effects in this category were mild (grade I/II) and resolved without intervention, with serious adverse events being rare.

In studies involving doses of stem cells equal to or greater than 1 million cells (1 × 10^6^ cells/kg or more), adverse events were more pronounced. For example, Oh et al. [17] and Nabavi et al. [18] reported a higher incidence of headaches, back pain, and fever across various routes, including IT and IM administration. Petrou et al. [21] documented adverse events such as arthralgia, injection site pain, and nausea in patients receiving both IT and IM injections. The Syková et al. [22] study noted that 15 ± 4.5 × 10^6^ BM‐MSCs per dose led to similar side effects, with most being mild to moderate. Serious adverse events were recorded, including respiratory issues and other complications, but these were often attributed to underlying conditions rather than the stem cell treatment itself. The routes of administration in this category also varied, with both IT and IM methods being commonly used. Overall, the high‐dose group experienced a range of mild to moderate side effects, with no significant treatment‐related toxicity reported.

Across all doses and routes of administration, headaches were the most frequently reported adverse event, particularly in the group receiving 1 million cells or more. Other common side effects included back pain, fever, and nausea. The relationship between adverse events and both the dose and the route of administration appears to be minimal, as most side effects were mild and transient, with serious adverse events being generally low and often unrelated to the stem cell therapy. The safety profile of stem cell administration remains favorable across different dosing regimens and methods of delivery, indicating that both the dose and the route of administration play a role in the overall safety and tolerability of the treatment.

### 3.8. Meta‐Analysis of Eligible 10 Included Studies

#### 3.8.1. Impact of Stem Cells Administration on ALSFRS in Patient With ALS

**Figure 4:**
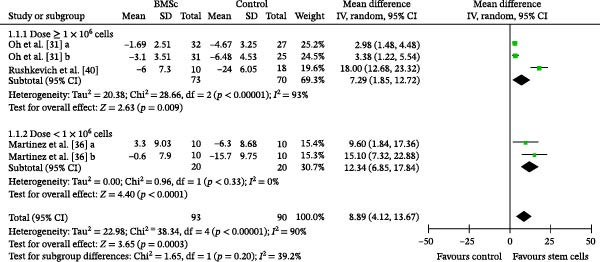
Forest plot illustrating the comparison of the Amyotrophic Lateral Sclerosis Functional Rating Scale (ALSFRS) scores between the stem cell treatment group and the control group among individuals with ALS. The analysis subgrouped by the dosage of stem cells administered. Oh et al. [31] indicate a follow‐up period of 4 months (a) and 6 months (b), while the findings from Martinez et al. [36] reflect follow‐up durations of 6 months (a) and 12 months (b).

**Figure 5:**
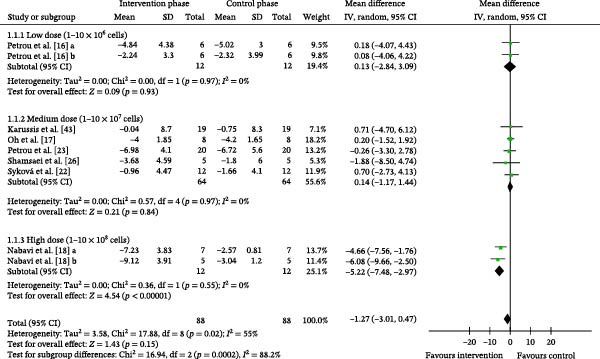
Amyotrophic Lateral Sclerosis Functional Rating Scale (ALSFRS) scores following a 3 ± 1 month intervention, subgrouped by applied dose (intrathecal, intramuscular, and intravenous). Petrou et al. [21] indicate the intrathecal route (a) and the intramuscular route (b). Additionally, the findings from Nabavi et al. [18] indicate the intrathecal route (a) and the intravenous route (b). Other studies also utilized the intrathecal route for their interventions.

This study included a meta‐analysis of three controlled trials and seven single‐arm studies to assess the ALSFRS in patients with ALS. Pooled analysis of three clinical trials with five outcomes (intervention: *n* = 52; control: *n* = 52) indicated significant slowed down the progression of ALSFRS in stem cell groups versus control groups (without transplantation, riluzole alone, standard symptomatic therapy [neurometabolites, anticonvulsants, antidepressants, etc.]) (WMD: 8.89; 95% CI: 4.12–13.67; *p* = 0.0003; *I* ^2^ = 90%), which was beneficial for both the ≥10 × 10^6^ and <10 × 10^6^ dose sub‐groups after 4, 6, and 12 weeks of intervention (WMD: 7.29; 95% CI: 1.85–12.72; *p*  < 0.00001; *I* ^2^ = 93%, and WMD: 12.34; 95% CI: 6.85–17.84; *p*  < 0.0001; *I* ^2^ = 0%, respectively) (Figure 4). In month 3 ± 1, a meta‐analysis of single‐arm studies (seven studies, nine outcomes, *n* = 88) in two control (pre‐intervention) and intervention phases demonstrated no significant difference in progression of ALSFRS between study phases via IT, IM, and IV routes (WMD: −1.27 (−3.01 to 0.47); *p* = 0.15) (Figure 5). Subgroup analysis by dosage revealed no significant difference in progression of ALSFRS between study phases for the low dose (1–10 × 10^6^; WMD: 0.13; 95% CI: −2.84 to 3.09; *p* = 0.93; *I* ^2^ = 0%), and medium dose (1–10 × 10^7^; WMD: 0.14; 95% CI: −1.14 to 1.44; *p* = 0.84; *I* ^2^ = 0%). However, in the high‐dose group, the progression of ALSFRS was significantly reduced during the intervention phases compared to the control (pre‐intervention) group (1–10 × 10^8^; WMD: −5.22; 95% CI: −7.48 to −2.97; *p*  < 0.00001; *I* ^2^ = 0%) (Figure 5).

**Figure 6:**
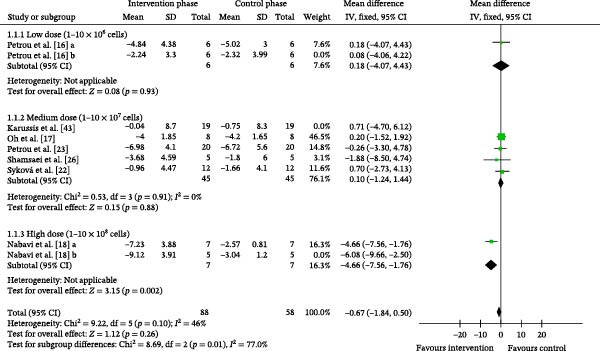
Amyotrophic Lateral Sclerosis Functional Rating Scale (ALSFRS) scores after a 3 ± 1 month intervention, subgrouped by applied dose. A sensitivity analysis was conducted by excluding the intramuscular and intravenous routes.

However, sensitivity analysis restricted to the IT delivery route (six studies, six outcomes, *n* = 58) indicated no significant differences between study phases (WMD: −0.67; 95% CI: −1.84 to 0.50; *p* = 0.26; *I* ^2^ = 46%) (Figure 6). The low (1–10 × 10^6^) and medium (1–10 × 10^7^) doses revealed no significant difference in progression of ALSFRS between study phases (WMD: 0.18; 95% CI: −4.07 to 4.43; *p* = 0.93, WMD: 0.10; 95% CI: −1.24 to 1.44; *p* = 0.88; *I* ^2^ = 0%, respectively). In the high‐dose group, the progression of ALSFRS was significantly reduced during the intervention phases compared to the control (pre‐intervention) group (1–10 × 10^8^; WMD: −4.66; 95% CI: −7.56 to −1.76; *p* = 0.002; *I* ^2^ = 0%) (Figure 6).

**Figure 7:**
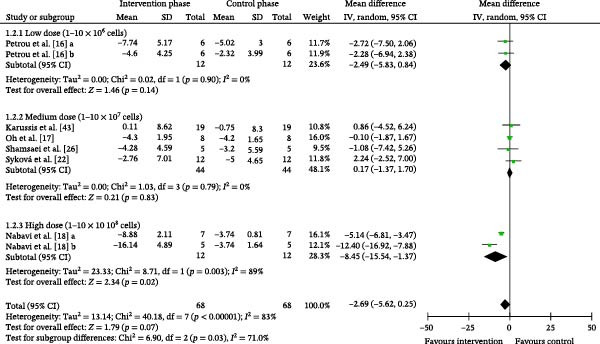
Amyotrophic Lateral Sclerosis Functional Rating Scale (ALSFRS) scores following a 6 ± 1 month intervention, subgrouped by applied dose (intrathecal, intramuscular, and intravenous). Petrou et al. [21] indicate the intrathecal route (a) and the intramuscular route (b). Additionally, the findings from Nabavi et al. [18] indicate the intrathecal route (a) and the intravenous route (b). Other studies also utilized the intrathecal route for their interventions.

In month 6 ± 1, a meta‐analysis of single‐arm studies (*n* = 68) in two pre‐intervention and intervention phases demonstrated no significant difference in progression of ALSFRS between study phases via IT, IM, and IV routes (WMD: −2.69; 95% CI: −5.62 to 0.25; *p* = 0.07, *I* ^2^ = 83%) (Figure 7). Subgroup analysis by dosage revealed no significant difference in progression of ALSFRS between study phases for the low dose (1–10 × 10^6^; WMD: −2.49; 95% CI: −5.83 to 0.84; *p* = 0.14; *I* ^2^ = 0%), and medium dose (1–10 × 10^7^; WMD: 0.17; 95% CI: −1.37 to 1.70; *p* = 0.83; *I* ^2^ = 0%). However, in the high‐dose group, the progression of ALSFRS was significantly reduced during the intervention phases compared to the pre‐intervention (1–10 × 10^8^; WMD: −8.48; 95% CI: −15.54 to −1.37; *p* = 0.003; *I* ^2^ = 89%) (Figure 7).

**Figure 8:**
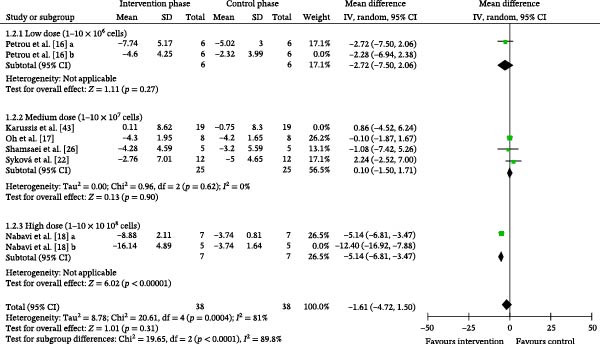
Amyotrophic Lateral Sclerosis Functional Rating Scale (ALSFRS) scores after a 6 ± 1 month intervention, subgrouped by applied dose. A sensitivity analysis was conducted by excluding the intramuscular and intravenous routes.

However, sensitivity analysis restricted to the IT delivery route (five studies, five outcomes, *n* = 38) indicated no significant differences between study phases (WMD: −1.61; 95% CI: −4.72 to 1.50; *p* = 0.31; *I* ^2^ = 81%). The low (1–10 × 10^6^) and medium (1–10 × 10^7^) doses revealed no significant difference in progression of ALSFRS between study phases (WMD: −2.72; 95% CI: −7.50 to 2.06; *p* = 0.27, WMD: 0.10; 95% CI: −1.50 to 1.71; *p* = 0.90; *I* ^2^ = 0%, respectively). In the high‐dose group, the progression of ALSFRS was significantly reduced during the intervention phases compared to the pre‐intervention (1–10 × 10^8^; WMD: −5.14; 95% CI: −6.81 to −3.47; *p*  < 0.00001) (Figure 8).

**Figure 9:**
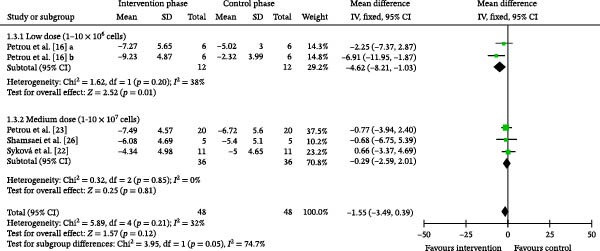
Amyotrophic Lateral Sclerosis Functional Rating Scale (ALSFRS) scores following a 9 ± 1 month intervention, subgrouped by applied dose (intrathecal, intramuscular, and intravenous). Petrou et al. [21] indicate the intrathecal route (a) and the intramuscular route (b). Other studies also utilized the intrathecal route for their interventions.

By month 9 ± 1, a meta‐analysis of single‐arm studies (*n* = 48) in two pre‐intervention and intervention phases demonstrated no significant difference in progression of ALSFRS between study phases via IT, IM, and IV routes (WMD: −1.55; 95% CI −3.49 to 0.39; *p* = 0.12, *I* ^2^ = 32%). Subgroup analysis by dosage revealed that in the low‐dose group, the progression of ALSFRS was significantly reduced during the intervention phases compared to the pre‐intervention (1–10 × 10^6^; WMD: −4.62; 95% CI: −8.21 to −1.03; *p* = 0.01; *I* ^2^ = 38%). However, no significant difference was obtained in progression of ALSFRS between study phases for the medium dose (1–10 × 10^7^; WMD: −0.29; 95% CI: −2.29 to 2.01; *p* = 0.81; *I* ^2^ = 0%) (Figure 9).

**Figure 10:**
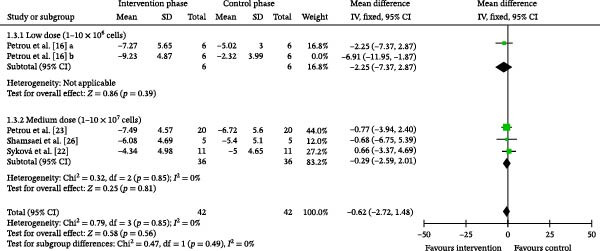
Amyotrophic Lateral Sclerosis Functional Rating Scale (ALSFRS) scores after a 9 ± 1 month intervention, subgrouped by applied dose. A sensitivity analysis was conducted by excluding the intramuscular and intravenous routes.

However, sensitivity analysis restricted to the IT delivery route (four studies, four outcomes, *n* = 42) indicated no significant differences between study phases (WMD: −0.62; 95% CI: −2.72 to 1.48; *p* = 0.56; *I* ^2^ = 0%). The low (1–10 × 10^6^) and medium (1–10 × 10^7^) doses revealed no significant difference in progression of ALSFRS between study phases (WMD: −2.25; 95% CI: −7.37 to 2.87; *p* = 0.39, WMD: −0.29; 95% CI: −2.59 to 2.01; *p* = 0.81; *I* ^2^ = 0%, respectively) (Figure 10).

**Figure 11:**
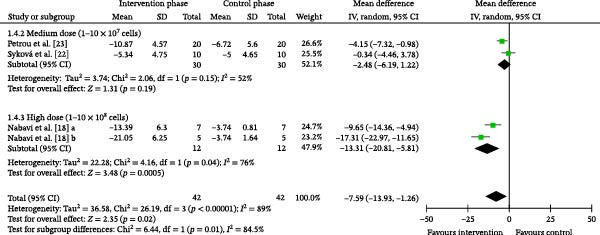
Amyotrophic Lateral Sclerosis Functional Rating Scale (ALSFRS) scores following a 12 ± 1 month intervention, subgrouped by applied dose (intrathecal, intramuscular, and intravenous). Nabavi et al. [18] indicate the intrathecal route (a) and the intravenous route (b). Other studies also utilized the intrathecal route for their interventions.

Finally, at month 12 ± 1, in a meta‐analysis of single‐arm studies (*n* = 42) in two pre‐intervention and intervention phases the progression of ALSFRS was significantly reduced during the intervention phases compared to the pre‐intervention via IT, IM, and IV routes (WMD: −7.59; 95% CI: −13.93 to −1.26; *p* = 0.02; *I* ^2^ = 89%). Subgroup analysis by dosage revealed that also in the high‐dose group, the progression of ALSFRS was significantly reduced during the intervention phases compared to the pre‐intervention (1–10 × 10^8^; WMD: −13.13; 95% CI: −20.81 to −5.81; *p* = 0.0005; *I* ^2^ = 76%). However, no significant difference was obtained in progression of ALSFRS between study phases for the low dose (1–10 × 10^6^; WMD: −2.84; 95% CI: −6.19 to 1.22; *p* = 0.19; *I* ^2^ = 52%) (Figure 11).

**Figure 12:**
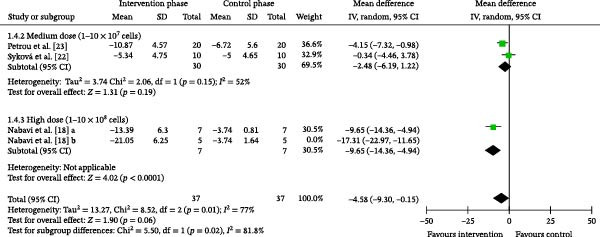
Amyotrophic Lateral Sclerosis Functional Rating Scale (ALSFRS) scores after a 12 ± 1 month intervention, subgrouped by applied dose. A sensitivity analysis was conducted by excluding the intramuscular and intravenous routes.

By deletion of IV rout in Nabavi et al. study, sensitivity analysis restricted to the IT delivery route (three studies, three outcomes, *n* = 37) indicated no significant differences between study phases (WMD: −4.58; 95% CI: −9.30 to 0.15; *p* = 0.06; *I* ^2^ = 77%). in the high‐dose group, the progression of ALSFRS was significantly reduced during the intervention phases compared to the pre‐intervention (1–10 × 10^8^; WMD: −9.65; 95% CI: −14.36 to −4.94; *p*  < 0.0001). However, no significant difference was obtained in progression of ALSFRS between study phases for the low dose (1–10 × 10^6^; WMD: −2.48; 95% CI: −6.19 to 1.22; *p* = 0.19; *I* ^2^ = 52%) (Figure 12).

#### 3.8.2. Impact of Stem Cells Administration on FVC in Patient With ALS

Two controlled trial studies were conducted to assess the impact of cell‐based therapies on FVC in ALS patients. In the first study by Martinez et al. [36], FVC in the treatment group receiving CD133+ stem cell transplantation ranged from 25% to 70%, with a mean of 44.6%, while the control group exhibited FVC values ranging from 20% to 80%, with a mean of 49.3%. However, statistical comparisons of FVC between the two groups at the 1‐year follow‐up could not be performed due to the presence of patients on ventilatory support and those who had died, ultimately showing no significant differences in FVC between the treatment and control groups. In the second study by Oh et al. [31] Repeated IT MSC for ALS, the effect of autologous BM‐MSCs on FVC was evaluated in a phase 2 RCT involving 64 participants. The results indicated that there were no significant differences in FVC between the MSC group and the control group during the 4‐month follow‐up period. This study concluded that while BM‐MSC treatment may have shown potential benefits in other efficacy outcomes, such as the ALS Functional Rating Scale (ALSFRS) scores, it did not lead to significant improvements in FVC measurements.

**Figure 13:**
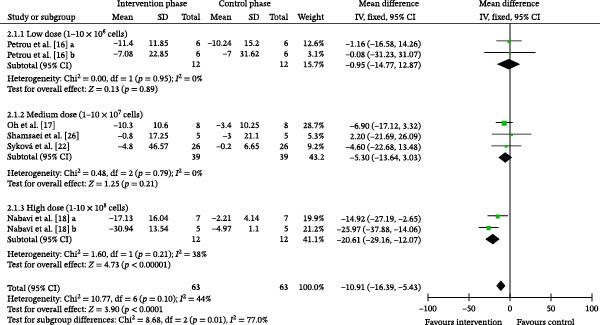
Forced vital capacity (FVC) scores following a 3 ± 1 month intervention, subgrouped by applied dose (intrathecal, intramuscular, and intravenous). Petrou et al. [21] indicate the intrathecal route (a) and the intramuscular route (b). Additionally, the findings from Nabavi et al. [18] indicate the intrathecal route (a) and the intravenous route (b). Other studies also utilized the intrathecal route for their interventions.

A pooled analysis of five single‐arm studies conducted at 3 ± 1 months (seven outcomes, *n* = 63) indicated that the rate of decline in FVC significantly increased during the intervention phase when compared to control pre‐intervention phase (administered via IT, IM, or IV routes) (WMD: −10.91; 95% CI: −16.39 to −5.43; *p*  < 0.0001; *I* ^2^ = 44%). Subgroup analysis by dosage revealed no significant difference in progression of FVC between study phases for the low dose (1–10 × 10^6^; WMD: −0.95; 95% CI: −14.77 to 12.87; *p* = 0.89; *I* ^2^ = 0%), and medium dose (1–10 × 10^7^; WMD: −5.30; 95% CI: −13.64 to 3.03; *p* = 0.21; *I* ^2^ = 0%). However, in the high‐dose group, the rate of decline in FVC significantly increased during the intervention phase when compared to control pre‐intervention phase (1–10 × 10^8^; WMD: −20.61; 95% CI: −29.16 to −12.07; *p*  < 0.00001; *I* ^2^ = 38%) (Figure 13)

**Figure 14:**
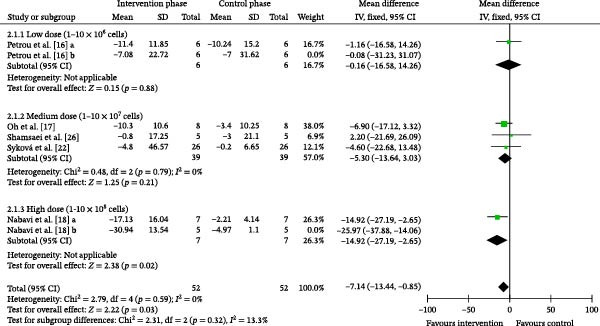
Forced vital capacity (FVC) scores after a 3 ± 1 month intervention, subgrouped by applied dose. A sensitivity analysis was conducted by excluding the intramuscular and intravenous routes.

Similarly, sensitivity analysis restricted to the IT delivery route (five studies, five outcomes, *n* = 52) indicated that the rate of decline in FVC significantly increased during the intervention phase when compared to control pre‐intervention phase (WMD: −7.14; 95% CI: −13.44 to −0.85; *p* = 0.03; *I* ^2^ = 0%). Subgroup analysis by dosage revealed no significant difference in progression of FVC between study phases for the low dose (1–10 × 10^6^; WMD: −1.16; 95% CI: −16.58 to 14.26; *p* = 0.88), and medium dose (1–10 × 10^7^; WMD: −5.30; 95% CI: −13.64 to 3.03; *p* = 0.21; *I* ^2^ = 0%). However, in the high‐dose group, the rate of decline in FVC significantly increased during the intervention phase when compared to control pre‐intervention phase (1–10 × 10^8^; WMD: −14.92; 95% CI: −27.19 to −2.65; *p* = 0.02) (Figure 14).

**Figure 15:**
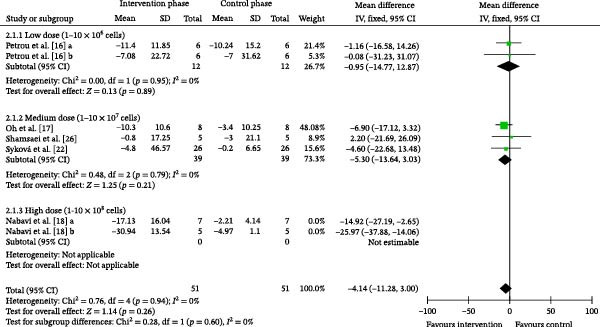
Sensitivity analysis conducted by excluding studies involving high‐dose stem cells after a 3 ± 1 month intervention.

The high dose (1–10 × 10^8^) in Nabavi et al. study at 3 ± 1 months had a significant impact on FVC, as sensitivity analysis by excluding high dose (five studies, five outcomes, *n* = 51) indicated no significant difference in progression of FVC between study phases (WMD: −4.14; 95% CI: −11.28 to 3.00; *p* = 0.26; *I* ^2^ = 0%). Subgroup analysis by dosage revealed no significant difference in progression of FVC between study phases for the low dose (1–10 × 10^6^; WMD: −0.95; 95% CI: −14.77 to 12.87; *p* = 0.89; *I* ^2^ = 0%), and medium dose (1–10 × 10^7^; WMD: −5.30; 95% CI: −13.64 to 3.03; *p* = 0.21; *I* ^2^ = 0%) (Figure 15).

**Figure 16:**
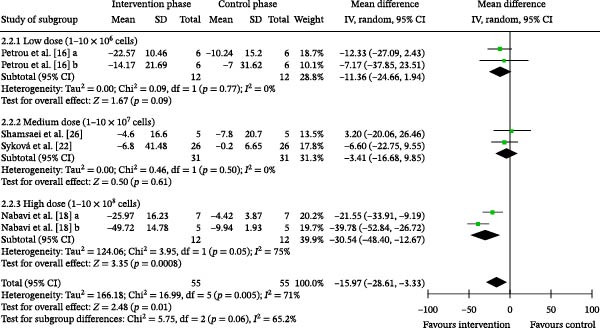
Forced vital capacity (FVC) scores following a 6 ± 1 month intervention, subgrouped by applied dose (intrathecal, intramuscular, and intravenous). Petrou et al. [21] indicate the intrathecal route (a) and the intramuscular route (b). Additionally, the findings from Nabavi et al. [18] indicate the intrathecal route (a) and the intravenous route (b). Other studies also utilized the intrathecal route for their interventions.

A pooled analysis of four single‐arm studies conducted at 6 ± 1 months (seven outcomes, *n* = 55) indicated that the rate of decline in FVC significantly increased during the intervention phase when compared to control pre‐intervention phase (administered via IT, IM, or IV routes) (WMD: −15.97; 95% CI: −28.61 to −3.33; *p* = 0.01; *I* ^2^ = 71%). Subgroup analysis by dosage revealed no significant difference in progression of FVC between study phases for the low dose (1–10 × 10^6^; WMD: −11.36; 95% CI: −24.66 to 1.94; *p* = 0.09; *I* ^2^ = 0%), and medium dose (1–10 × 10^7^; WMD: −3.41; 95% CI: −16.68 to 9.85; *p* = 0.61; *I* ^2^ = 0%). However, in the high‐dose group, the rate of decline in FVC significantly increased during the intervention phase when compared to control pre‐intervention phase (1–10 × 10^8^; WMD: −30.54; 95% CI: −48.40 to −12.67; *p* = 0.0008; *I* ^2^ = 75%) (Figure 16).

**Figure 17:**
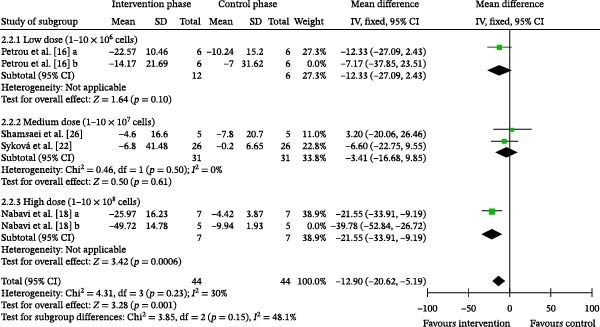
Forced vital capacity (FVC) scores after a 6 ± 1 month intervention, subgrouped by applied dose. A sensitivity analysis was conducted by excluding the intramuscular and intravenous routes.

Similarly, sensitivity analysis restricted to the IT delivery route at 6 ± 1 months (4 studies, 4 outcomes, *n* = 44) indicated that the rate of decline in FVC significantly increased during the intervention phase when compared to control pre‐intervention phase (WMD: −12.90; 95% CI: −20.62 to −5.19; *p* = 0.001; *I* ^2^ = 30%). Subgroup analysis by dosage revealed no significant difference in progression of FVC between study phases for the low dose (1–10 × 10^6^; WMD: −12.33; 95% CI: −27.09 to 2.43; *p* = 0.10), and medium dose (1–10 × 10^7^; WMD: −3.41; 95% CI: −16.68 to 9.85; *p* = 0.61; *I* ^2^ = 0%). However, in the high‐dose group, the rate of decline in FVC significantly increased during the intervention phase when compared to control pre‐intervention phase (1–10 × 10^8^; WMD: −21.55; 95% CI: −33.91 to −9.19; *p* = 0.0006) (Figure 17).

**Figure 18:**
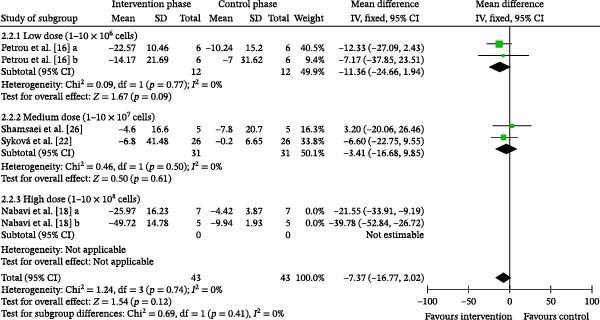
Sensitivity analysis conducted by excluding studies involving high‐dose stem cells after a 6 ± 1 month intervention.

The high dose (1–10 × 10^8^) in the Nabavi et al. [18] also at 6 ± 1 months revealed a significant effect on FVC, indicating that such high doses do not lead to positive effect for FVC, as sensitivity analysis by excluding high dose (four studies, four outcomes, *n* = 43) indicated no significant difference in progression of FVC between study phases (WMD: −7.37; 95% CI: −16.77 to 2.02; *p* = 0.12; *I* ^2^ = 0%). Subgroup analysis by dosage revealed no significant difference in progression of FVC between study phases for the low dose (1–10 × 10^6^; WMD: −11.36; 95% CI: −24.66 to 1.94; *p* = 0.09; *I* ^2^ = 0%), and medium dose (1–10 × 10^7^; WMD: −3.41; 95% CI: −16.68 to 9.85; *p* = 0.61; *I* ^2^ = 0%) (Figure 18).

**Figure 19:**
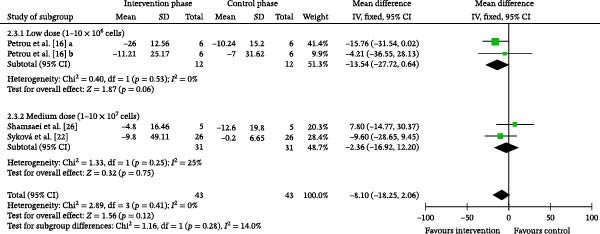
Forced vital capacity (FVC) scores following a 9 ± 1 month intervention, subgrouped by applied dose (intrathecal, intramuscular, and intravenous). Petrou et al. [21] indicate the intrathecal route (a) and the intramuscular route (b). Other studies also utilized the intrathecal route for their interventions.

At 9 ± 1 months, a pooled analysis of three single‐arm studies (four outcomes, *n* = 43) no significant difference in progression of FVC between study phases (administered via IT, IM, or IV routes) (WMD: −8.10; 95% CI: −18.25 to 2.03; *p* = 0.12; *I* ^2^ = 0%). Subgroup analysis by dosage also revealed no significant difference in progression of FVC between study phases for the low dose (1–10 × 10^6^; WMD: −13.54; 95% CI: −27.72 to 0.64; *p* = 0.06; *I* ^2^ = 0%), and medium dose (1–10 × 10^7^; WMD: −2.36; 95% CI: −16.92 to 12.20; *p* = 0.75; *I* ^2^ = 25%). The lack of significant findings at 9 months, as opposed to the results seen in months three and six, can be attributed to the absence of the Nabavi et al. [18] study in this time period (the 9‐month assessment had not been conducted in this study) (Figure 19).

**Figure 20:**
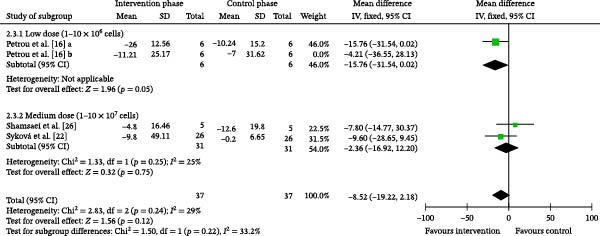
Forced vital capacity (FVC) scores after a 9 ± 1 month intervention, subgrouped by applied dose. A sensitivity analysis was conducted by excluding the intramuscular and intravenous routes.

Similarly, sensitivity analysis restricted to the IT delivery route at 9 months (three studies, four outcomes, *n* = 37) indicated no significant difference in progression of FVC between study phases (WMD: −8.52; 95% CI: −19.22 to 2.18; *p* = 0.12; *I* ^2^ = 29%). Subgroup analysis by dosage also revealed no significant difference in progression of FVC between study phases for the low dose (1–10 × 10^6^; WMD: −15.76; 95% CI: −31.54 to 0.02; *p* = 0.05), and medium dose (1–10 × 10^7^; WMD: −2.36; 95% CI: −16.92 to 12.20; *p* = 0.75; *I* ^2^ = 25%) (Figure 20).

## 4. Discussion

This systematic review and meta‐analysis evaluated the efficacy of stem cell therapy in managing ALS. A total of 31 studies (7 controlled trials and 24 non‐controlled studies) with 830 participants were included. A pooled analysis of controlled trials (*n* = 183) demonstrated that stem cell therapy significantly slowed the functional decline of ALS patients, as measured by a reduced progression of ALSFRS scores compared to controls. An analysis of single‐arm studies comparing to their pre‐intervention phases (*n* = 88) showed no significant difference in ALSFRS score progression at months 3, 6, and 9. A significant difference was observed only at month 12. The rate of decline in FVC significantly increased at months 3 and 6 in the intervention comparing the control pre‐intervention phase. However, sensitivity analysis by removing the high‐dose MSCs therapies indicated no significant differences between study phases. Adverse events were mostly mild, with headaches being most frequent in high‐dose groups.

ALS is a progressive neurodegenerative disorder characterized by the degeneration of motor neurons in the brain and spinal cord, leading to muscle weakness, atrophy, and ultimately respiratory failure. Despite significant advances in understanding the pathophysiology of ALS, effective treatments remain limited. Recent studies have explored the potential of stem cell therapy as a novel approach to mitigate the progression of ALS and improve patient outcomes.

The mechanisms underlying the potential efficacy of stem cell therapy in ALS are multifaceted. First, stem cells, particularly MSCs, exhibit neuroprotective effects by secreting neurotrophic factors such as brain‐derived neurotrophic factor (BDNF) and glial cell‐derived neurotrophic factor (GDNF), which promote motor neuron survival and function [51, 52]. Additionally, stem cells possess immunomodulatory properties that can help reduce neuroinflammation, a key contributor to motor neuron degeneration in ALS, by modulating the activation of microglia and astrocytes [53–55]. Furthermore, stem cells may facilitate regeneration and repair by differentiating into various cell types, including neurons and glial cells, thereby supporting endogenous repair mechanisms [56]. They also have the potential to enhance angiogenesis, improving blood flow to affected areas and promoting neuronal health [57]. While clinical trials have reported varying degrees of efficacy, with some studies showing improvements in functional outcomes such as ALSFRS‐R scores and FVC, challenges remain regarding standardized protocols for cell sourcing, dosing, and administration routes [13]. Understanding the long‐term effects and potential risks associated with stem cell therapy is crucial for its safe application in ALS patients.

A pooled analysis of three clinical trials with five outcomes indicated that stem cell therapy significantly reduced the progression of ALSFRS scores in ALS patients. This was beneficial for both the ≥10 × 10^6^ and <10 × 10^6^ dose sub‐groups. An analysis of single‐arm studies comparing to their pre‐intervention phases (*n* = 88) showed no significant difference in ALSFRS score progression at months 3, 6, and 9. A significant difference was observed only at month 12.

The effect of stem cell therapy on the ALSFRS in ALS patients has shown some promise, with certain studies reporting modest improvements in functional abilities. These improvements may reflect neuroprotective effects and the potential for motor neuron survival, which could slow the disease progression [58]. However, clinical trial results have been mixed, with variability in outcomes depending on factors such as the type of stem cells used, the method of administration, and the timing of treatment. While some patients have experienced enhancements in their ALSFRS scores, the optimal dosing of stem cells remains unclear [59] as higher doses may lead to better outcomes in some cases, while lower doses can also be effective. Overall, while there is potential for stem cell therapy to positively impact ALSFRS scores, further research is needed to establish its efficacy and long‐term benefits.

While some studies show promising results with increased cell doses and repeated injections [23], outcomes depend heavily on individual patient characteristics. The research indicates that patient selection, injection frequency, and individual disease progression rates are potentially more critical than absolute stem cell dosage [29], demonstrated that escalating doses can be safely administered, but did not conclusively prove that higher doses directly translate to better clinical outcomes.

Regarding FVC, this review indicated that high‐dose stem cell therapy, particularly at 1–10 × 10^8^ cells, is associated with a significantly accelerated decline in FVC in ALS patients. In contrast, low and medium doses showed no significant negative impact on FVC progression. This demonstrates that high‐dose administration significantly reduces respiratory capacity.

Multiple studies indicate that while high‐dose MSC therapy is generally well‐tolerated [60, 61], it poses significant respiratory risks in patients with ALS. Specifically, high doses or rapid infusion rates can activate coagulation, increase plasma viscosity, and trigger thrombus formation in pulmonary vessels [62, 63] mechanisms that may be exacerbated in ALS patients with underlying microvascular lung impairment. Although reports of respiratory failure and mortality following stem cell transplantation exist [38, 64], a direct causal link to lung failure has not been definitively established. However, the progressive respiratory dysfunction and weakened airway defenses in ALS [65] heighten vulnerability to such complications. To mitigate these risks, careful monitoring of infusion rates, use of lower doses, and pre‐screening for thrombosis risk factors are essential [66, 67].

In a comprehensive systematic review, Zhang et al. [68] found that neural stem/progenitor cell transplantation demonstrated potential positive effects on FVC in ALS patients. Specifically, their meta‐analysis of 16 investigations (including 5 clinical studies and 11 animal studies) revealed modest improvements in physical function, including FVC measurements. Conversely, a thorough meta‐analysis by Morata‐Tarifa et al. [13], which included 220 patients from 11 studies, revealed that although IT mesenchymal stromal cell injections provided temporary clinical improvements, all interventions ultimately led to a decline in respiratory function. However, the evidence remains tentative. Aljabri et al. [69] concluded that while some studies showed positive effects of stem cell transplantation, others found no significant difference compared to control groups. Moura et al. [12] further emphasized that clinical studies are still insufficient to definitively assess effectiveness, with current research only demonstrating the absence of serious adverse events.

However, this review reveals several significant limitations that impact the interpretation of the findings. First, the majority of included studies were non‐controlled pre–post studies, which introduce bias and limit the ability to draw definitive conclusions about the efficacy of the treatments. Second, there is considerable heterogeneity in the types of stem cells used, the dosing regimens, inconsistent follow‐up duration, and variability in stem cell preparation, making it challenging to compare results across studies and determine optimal treatment protocols. Lastly, the short follow‐up duration in many studies limit the understanding of the long‐term effects and potential risks associated with stem cell therapy, underscoring the need for more rigorous, controlled research to establish the efficacy and safety of these interventions in ALS patients.

## 5. Conclusion

Stem cell therapy for ALS appears to be safe, with preliminary evidence suggesting that stem cell therapy may attenuate the progression of ALS, as shown by improved ALSFRS scores in controlled trials. However, two‐phase single‐arm studies indicated no significant differences between the treatment and pre‐treatment phases. Therefore, the existing evidence base remains exploratory, and definitive conclusions regarding clinical effectiveness cannot yet be drawn. Moreover, while some studies suggested transient slowing of disease progression or possible survival benefits, these findings were often short‐lived and inconsistently replicated across studies. Robust evidence demonstrating sustained functional improvement, long‐term respiratory stabilization, or significant survival extension remains lacking. Future research should prioritize large‐scale, multicenter, RCTs with standardized cell manufacturing protocols, longer follow‐up periods, validated biomarkers, and stratification of patient subgroups to determine the optimal stem cell type, route of administration, dosing strategy, and therapeutic timing for ALS treatment.

**Nomenclature**
ALS: Amyotrophic lateral sclerosis
RCTs: Randomized controlled trials
ALSFRS: Amyotrophic Lateral Sclerosis Functional Rating Scale
FVC: Forced vital capacity
IT: Intrathecal
IM: Intramuscular
IV: Intravenous
MNDs: Motor neuron diseases
SMA: Spinal muscular atrophy
NMDs: Neuromuscular diseases
DMD: Duchenne muscular dystrophy.

## Author Contributions

Mahdieh Pourasghari, Bina Eftekharsadat, and Azizeh Farshbaf‐Khalili were responsible for the conceptualization. Mahdieh Pourasghari, Soraya Babaie, Raha Markazi‐Movaghar, Saba Salehpour, Bina Eftekharsadat, and Azizeh Farshbaf‐Khalili contributed to the methodology. Azizeh Farshbaf‐Khalili and Soraya Babaie handled the software aspects. Mahdieh Pourasghari, Soraya Babaie, Bina Eftekharsadat, and Azizeh Farshbaf‐Khalili conducted the validation. Soraya Babaie and Azizeh Farshbaf‐Khalili performed formal analysis. Mahdieh Pourasghari, Soraya Babaie, Bina Eftekharsadat, and Azizeh Farshbaf‐Khalili managed data curation. Saba Salehpour prepared original draft, while Azizeh Farshbaf‐Khalili, Soraya Babaie, Raha Markazi‐Movaghar, and Bina Eftekharsadat were involved in writing the review and editing. Bina Eftekharsadat did the visualization. Bina Eftekharsadat and Azizeh Farshbaf‐Khalili provided the supervision. Azizeh Farshbaf‐Khalili oversaw the project administration.

## Funding

This study was supported by the University of Medical Sciences, Tabriz, Iran (Grant 74832).

## Disclosure

All authors have seen and approved the final version of the manuscript being submitted. They warrant that the article is the authors’ original work, has not received prior publication, and is not under consideration for publication elsewhere.

## Conflicts of Interest

The authors declare no conflicts of interest.

## Supporting Information

Additional supporting information can be found online in the Supporting Information section.

## Supporting information


**Supporting Information** Supporting Information contains the detailed search strategies for PubMed and Embase used in this review.

## Data Availability

Data are available upon request from the authors.
