# Supplementary material for: The Effectiveness Based on Optimal Dose and Administration Route, and Safety Profiles of Stem Cells and Derived Products in the Treatment of Patients With Amyotrophic Lateral Sclerosis: A Systematic Review and Meta‐Analysis
Source: Stem Cells Int. 2026 Aug 5;2026:4122493. doi: 10.1155/sci/4122493 (PMC13439401; doi:10.1155/sci/4122493)
Supplement: Supplementary file 1 [file SCI-2026-4122493-s001.docx]

**Supplementary material 1.** Search strategy

Embase

('lower motor neuron weakness':ti,ab OR 'weakness'/exp OR 'neuromuscular weakness':ti,ab OR 'congenital structural myopathies':ti,ab OR 'myopathy'/exp OR 'hereditary myopathy':ti,ab OR 'myopathies' OR 'spinal muscular atrophy' OR 'amyotrophic lateral sclerosis'/exp OR 'progressive muscular atrophy':ti,ab OR 'motor neuron disease':ti,ab) AND ('stem cells':ti,ab OR 'exosomes':ti,ab OR 'secretome') AND 'clinical trial':ti,ab

Pubmed

(('Stem Cells'[Mesh]) OR 'Exosomes'[Mesh]) OR 'Secretome'[Mesh]) OR ('exosome'[Text Word] OR 'secretome'[Text Word] OR 'stem cell'[Text Word])) AND ('lower motor neuron weakness'[Text Word] OR 'weakness'[Text Word] OR 'neuromuscular weakness'[Text Word] OR 'congenital structural myopathies'[Text Word] OR 'myopathy'[Text Word] OR 'hereditary myopathy'[Text Word] OR 'myopathies'[Text Word] OR 'spinal muscular atrophy'[Text Word] OR 'Amyotrophic lateral sclerosis'[Text Word] OR 'Progressive muscular atrophy'[Text Word] OR 'motor neuron disease'[Text Word])) AND ('clinical trial'[Text Word] OR 'trial'[Text Word] OR 'clinical'[Text Word])
